# Supplementary figures and images for: Effect of mupirocin for Staphylococcus aureus decolonization on the microbiome of the nose and throat in community and nursing home dwelling adults
Source: PLoS One. 2021 Jun 8;16(6):e0252004. doi: 10.1371/journal.pone.0252004 (PMC8186807; doi:10.1371/journal.pone.0252004)

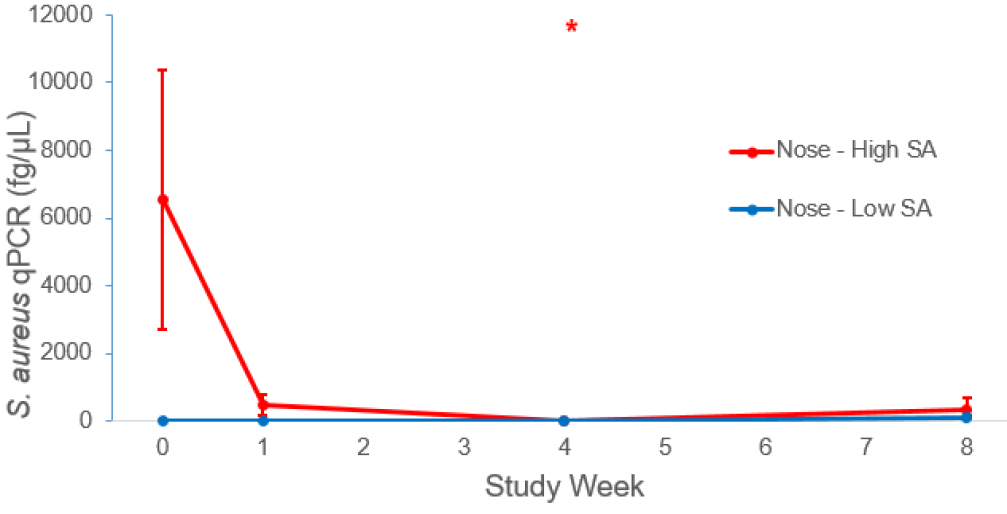

Supplement: S1 Fig — Data are presented as means ± SEMs. Statistical comparisons were made with the Week 0 time point within SA group. Statistical significance was determined by Wilcoxon signed ranks test. Asterisk, P<0.05. (TIF) [file pone.0252004.s002.tif]

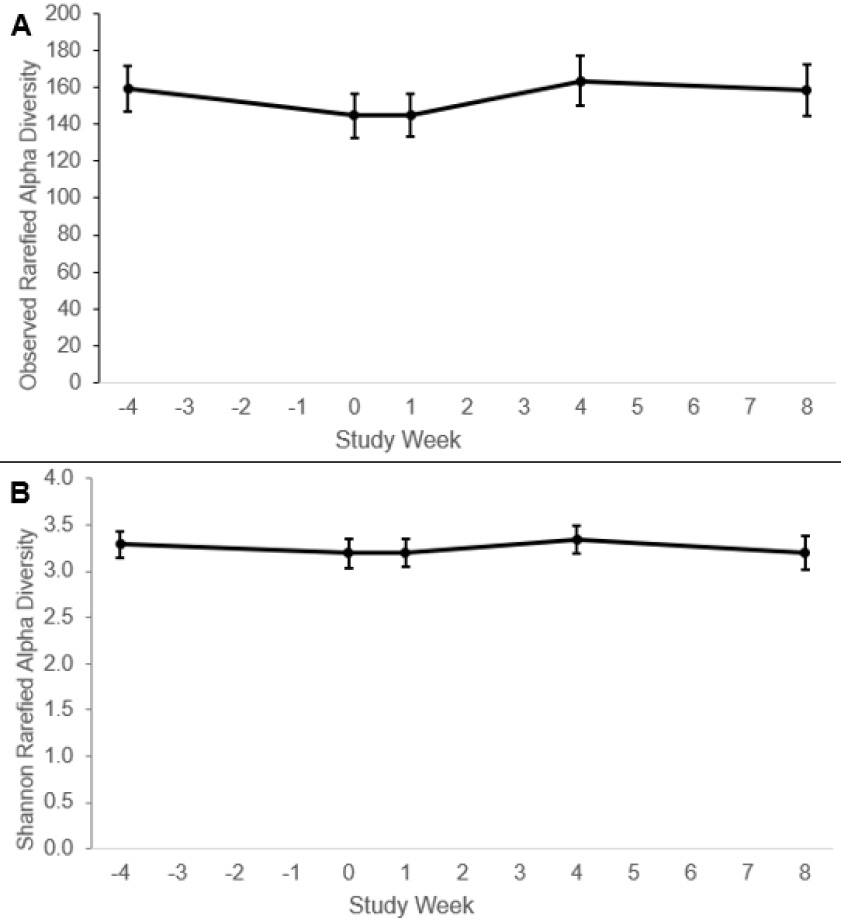

Supplement: S2 Fig — A. Observed Diversity Index, B. Shannon Diversity Index. Data are presented as means ± SEMs. Statistical comparisons were made with the Week 0 time point. Statistical significance was determined by Wilcoxon rank sum test (Mann Whitney U test). Asterisk, P<0.05. (TIF) [file pone.0252004.s003.tif]

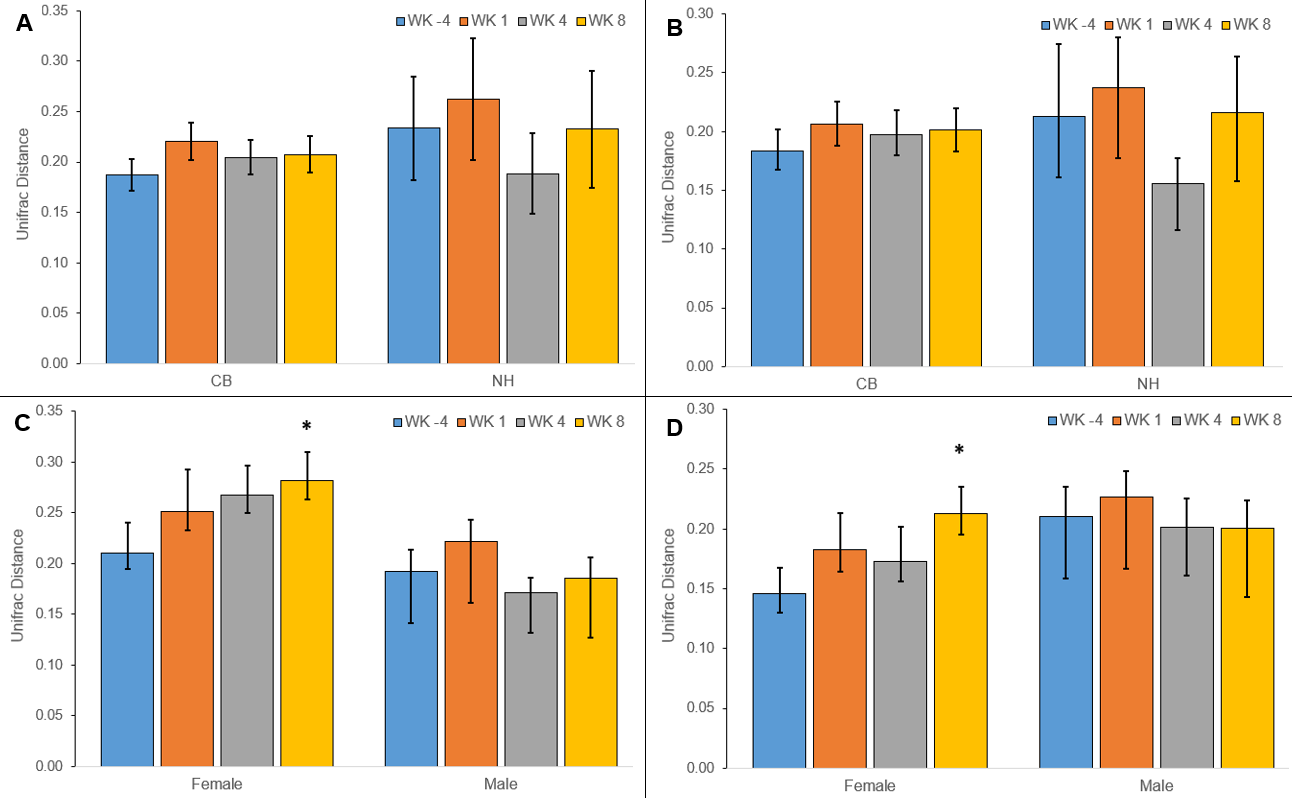

Supplement: S3 Fig — Unifrac distances comparing distances to week 0 communities by body site stratified by dwelling (A: nose, B: throat) and gender (C: nose, D: throat). Data are presented as means ± SEMs. Statistical comparisons were made between the distance of Week -4 to Week 0, and the distance of Week 0 to other time points within body site. Statistical significance was determined by Wilcoxon signed ranks test. Asterisk, P<0.05. (TIF) [file pone.0252004.s004.tif]

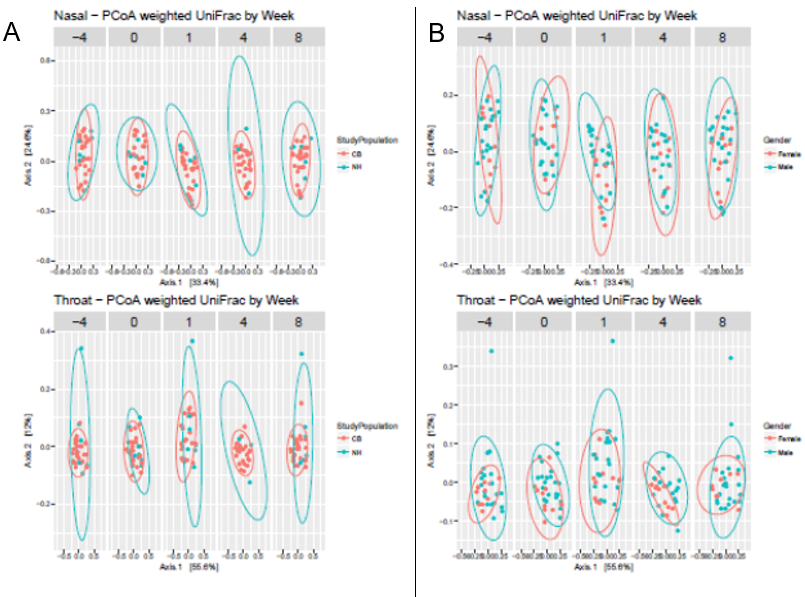

Supplement: S4 Fig — Principal coordinates (PCs) analysis of beta diversity metrics by body site stratified by dwelling (A) and gender (B), showing distances from unweighted Unifrac over time. The ellipses represent 95% confidence intervals for clustered specimens from participants by gender and dwelling. (TIF) [file pone.0252004.s005.tif]

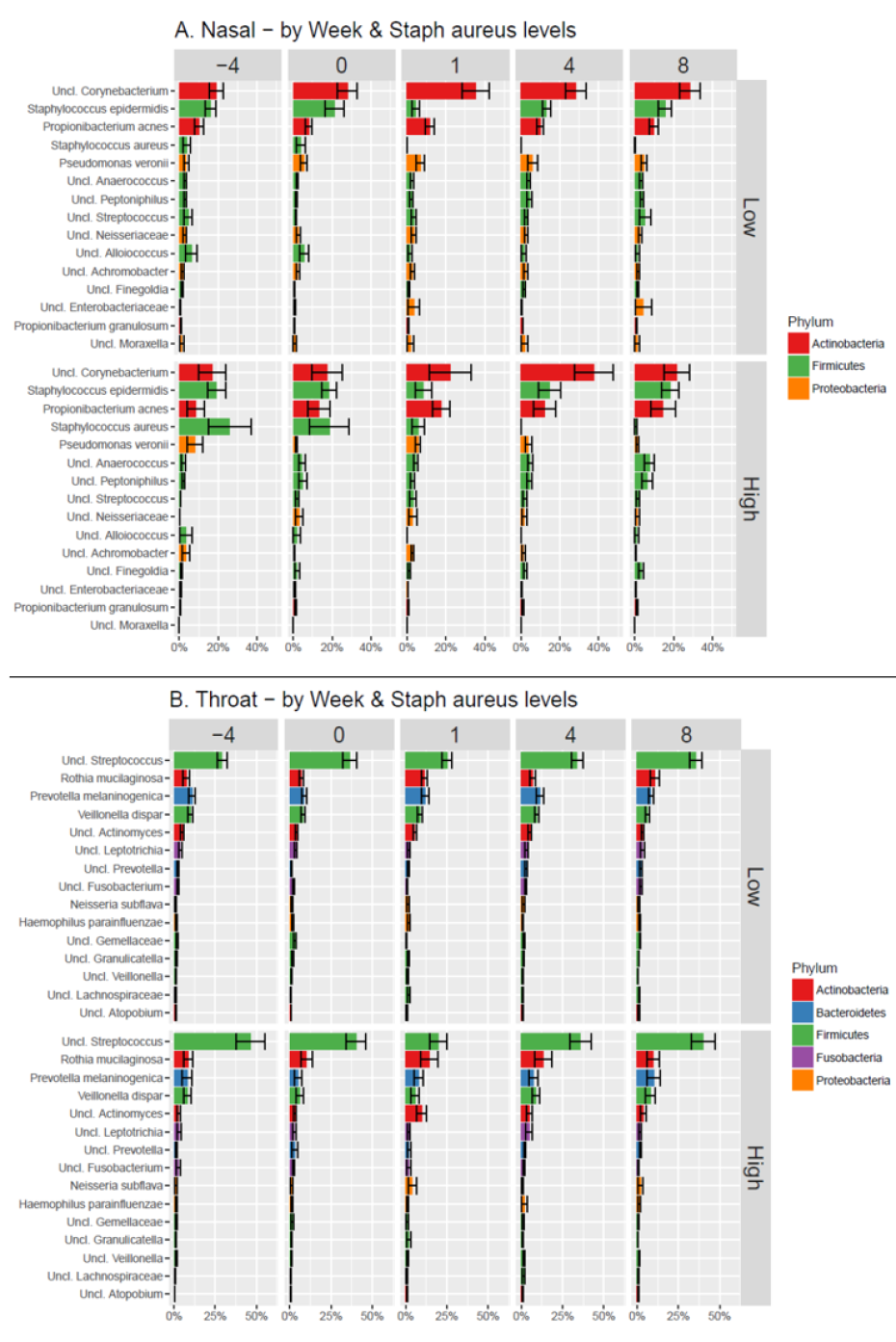

Supplement: S5 Fig — Error bars show standard errors. A. Nose, B. Throat. (TIF) [file pone.0252004.s006.tif]

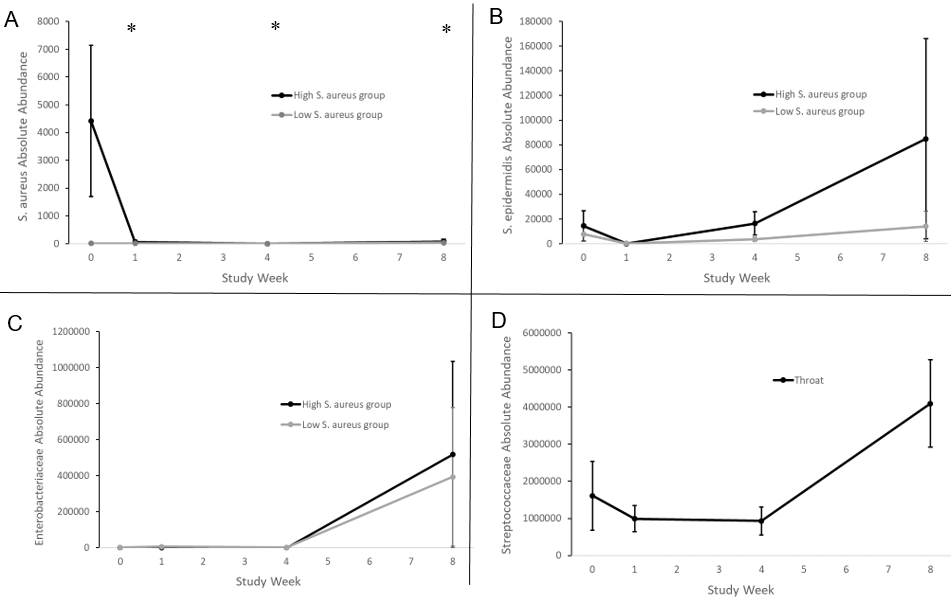

Supplement: S6 Fig — Data are presented as means ± SEMs. Statistical comparisons were made between participants with high and low relative abundance of Staphylococcus aureus of the change from Week 0 to each time point within the nose (A, B, C) or of the change from Week 0 to each time point within the throat (D). Statistical significance was determined by Wilcoxon rank sum test (Mann Whitney U test) (A, B, C) or the Wilcoxon signed ranks test (D). Asterisk, P<0.05. (TIF) [file pone.0252004.s007.tif]
