# Supplementary material for: Effect of mupirocin for Staphylococcus aureus decolonization on the microbiome of the nose and throat in community and nursing home dwelling adults
Source: PLoS One. 2021 Jun 8;16(6):e0252004. doi: 10.1371/journal.pone.0252004 (PMC8186807; doi:10.1371/journal.pone.0252004)
Supplement: S1 Table — (DOCX) [file pone.0252004.s008.docx]

S1 Table – Differentially abundant bacteria in the nose and throat over time using generalized linear models of abundance based on the negative binomial distribution. ^1^

| **Body site** | **Comparison** | **OTUID** | **log2FoldChange** | **P value** | **Adjusted P value** |
| --- | --- | --- | --- | --- | --- |
| Nose | Week 1 vs 0 | Staphylococcus epidermidis (2) | -3.03 | 0.00000 | 0.00000 |
| Nose | Week 1 vs 0 | Staphylococcus aureus (11634) | -5.11 | 0.00000 | 0.00000 |
| Nose | Week 1 vs 0 | Uncl. Propionibacterium (13109) | -4.73 | 0.00000 | 0.00017 |
| Nose | Week 1 vs 0 | Staphylococcus epidermidis (3808) | -3.88 | 0.00000 | 0.00031 |
| Nose | Week 1 vs 0 | Staphylococcus epidermidis (7625) | -4.39 | 0.00001 | 0.00031 |
| Nose | Week 1 vs 0 | Uncl. Corynebacterium (10047) | -4.28 | 0.00003 | 0.00136 |
| Nose | Week 1 vs 0 | Staphylococcus haemolyticus (6316) | -3.26 | 0.00014 | 0.00498 |
| Throat | Week 1 vs 0 | Uncl. Gemellaceae (21) | -3.28 | 0.00000 | 0.00346 |
| Throat | Week 1 vs 0 | Staphylococcus epidermidis (2) | -2.55 | 0.00002 | 0.00623 |
| Throat | Week 1 vs 0 | Uncl. Streptococcus (10972) | -2.81 | 0.00004 | 0.00901 |
| Nose | Week 8 vs 0 | Staphylococcus aureus (11634) | -8.67 | 0.00000 | 0.00000 |
| Nose | Week 8 vs 0 | Uncl. Haemophilus (47) | 5.25 | 0.00000 | 0.00183 |
| Throat | Week 8 vs 0 | Staphylococcus aureus (11634) | -4.80 | 0.00000 | 0.00002 |

Reference

1. Love MI, Huber W, Anders S. Moderated estimation of fold change and dispersion for RNA-seq data with DESeq2. *Genome Biol* 2014; **15**: 550.
