# Supplementary material for: Effect of mupirocin for Staphylococcus aureus decolonization on the microbiome of the nose and throat in community and nursing home dwelling adults
Source: PLoS One. 2021 Jun 8;16(6):e0252004. doi: 10.1371/journal.pone.0252004 (PMC8186807; doi:10.1371/journal.pone.0252004)
Supplement: S1 File — (PDF) [file pone.0252004.s009.pdf]

PROTOCOL NAME
Metagenomic Studies of MRSA Colonization - Nose, Throat, and Skin Cultures before and after use of Antibacterial Nasal Ointment and Antibacterial Soap for Showering in Adults who live in the Community and have Current *S. aureus* Colonization
Short title: Cultures before and after Decolonization in Community Dwelling Adults with Current *S. aureus* Colonization

Grant: Metagenomic Studies of MRSA Colonization (VA Merit 1I01CX000491-01A1)

PROTOCOL IDENTIFYING NUMBER (any amendments should bear the amendment number)
CICERO protocol number: HP-50442

PROTOCOL VERSION DATE: January 9, 2020

Commonly Used Abbreviations

S. aureus	*Staphylococcus aureus*
MRSA	Methicillin-resistant *S. aureus*
MSSA	Methicillin-susceptible *S. aureus*
VAMHCS R&D	VA Maryland Health Care System Research and Development
UMB IRB	University of Maryland Baltimore Institutional Review Board

GENERAL INFORMATION

Name and address of the study monitor and person authorized to sign the protocol and amendments:

Mary-Claire Roghmann, MD, MS
Hospital Epidemiologist, Staff Physician and Research Health Scientist
Head, Epidemiology Section
Medical Care Clinical Center
VA Maryland Health Care System

Professor of Epidemiology and Public Health and Medicine
University of Maryland School of Medicine
685 W. Baltimore St., MSTF 3-36
Baltimore, MD 21201

Phone	(410) 706-0062
Fax	(410) 706-0098
Beeper	(410) 447-4143
E-mail	mroghman@epi.umaryland.edu

Name and addresses of the laboratories, study sites and/or other institutions involved in the study:

Microbiology Research Lab
Department of Pathology
Medical School Teaching Facility, Room 957
10 S. Pine St.
Baltimore, MD 21201

Institute for Genome Sciences (IGS)
University of Maryland School of Medicine
801 W Baltimore St
BioPark II, 6th Floor
Baltimore, MD 21201

Baltimore VA Medical Center
Microbiology Research Lab, 3B-112
Managed Care Clinics
10 North Greene Street
Baltimore, MD 21201

Table of Contents

PROTOCOL SUMMARY 4

1 SCHEMATIC OF STUDY DESIGN 5

2 BACKGROUND INFORMATION AND RATIONALE 6

2.1 BACKGROUND INFORMATION 6
2.2 RATIONALE 7
2.3 RISK/BENEFITS 7
2.4 STUDY CONDUCT 9

3 STUDY OBJECTIVES 9

4 STUDY DESIGN 9

4.1 PRIMARY STUDY ENDPOINTS/SECONDARY ENDPOINTS 9
4.2 STUDY DESIGN/TYPE 9
4.3 DURATION 10

5 SELECTION AND WITHDRAWAL OF PARTICIPANTS 10

5.1 SELECTION OF STUDY POPULATION 10
5.2 INCLUSION CRITERIA 10
5.3 EXCLUSION CRITERIA 10
5.4 PARTICIPANT WITHDRAWAL 11
5.5 MEDICATION 11

6 INFORMED CONSENT PROCESS FOR PARTICIPANTS 11

7 STUDY INTERVENTION 11

7.1 STUDY PRODUCT DESCRIPTION 11
7.1.1 Acquisition 11
7.1.2 Formulation, Packaging, and Labeling 12
7.1.3 Product Storage and Stability 12
7.2 DOSAGE, PREPARATION AND ADMINISTRATION OF STUDY INTERVENTION 12
7.3 MODIFICATION OF STUDY INTERVENTION 12
7.4 ACCOUNTABILITY PROCEDURES FOR THE STUDY INTERVENTION 12
7.5 ASSESSMENT OF PARTICIPANT COMPLIANCE WITH STUDY INTERVENTION 12

8 STUDY PROCEDURES/EVALUATIONS 13

8.1 CLINICAL EVALUATIONS 13
8.2 LABORATORY EVALUATIONS 16

9 UNANTICIPATED PROBLEMS AND SERIOUS ADVERSE EVENTS 16

10 QUALITY CONTROL AND QUALITY ASSURANCE 17

11 STATISTICAL PLAN 17

11.1 ANALYSIS PLAN FOR MICROBIAL COMMUNITY PROFILING 17
11.2 ANALYSIS PLAN FOR MICROBIOLOGIC DATA 18
11.3 SAMPLE SIZE ESTIMATES 18

12 ETHICAL CONSIDERATIONS 19

13 DATA HANDLING AND RECORD KEEPING 19

14 REFERENCES 21

Protocol Summary

Title: Metagenomic Studies of MRSA Colonization - Nose, Throat, and Skin Cultures before and after use of Antibacterial Nasal Ointment and Antibacterial Soap for Showering in Adults who live in the Community and have Current *S. aureus* Colonization

Population: Approximately 250 male and female adults with *S. aureus* colonization at enrollment from a single geographic location in the US to achieve a final sample size of at least 40.

Site: Baltimore VA Medical Center

Study Duration: Approximately 18 months

Duration of Participant's Participation: Up to 52 weeks

Objectives:

- To collect specimens from multiple body sites in a non-invasive manner to serve as sources of measurement of the core microbiomes associated with the anterior nares, posterior pharynx, skin over the right subclavian vein and the right femoral vein and perirectal skin (the skin around the anus), in *S. aureus* colonized adults
- To collect specimens from multiple body sites in a non-invasive manner to serve as sources of measurement of the change in the core microbiomes associated with the anterior nares, posterior pharynx and skin over the right subclavian vein and the right femoral vein and the perirectal skin in response to application of a nasal and skin *S. aureus* decolonization regimen in *S. aureus* colonized adults
- To collect serum from the participants to examine immune responses to organisms that are identified in the microbiome
- To collect blood from the participants to examine the relationship of host genotype (by either genotyping or sequencing the DNA) to organisms that are identified in the microbiome present on/in an individual

1 Schematic of Study Design

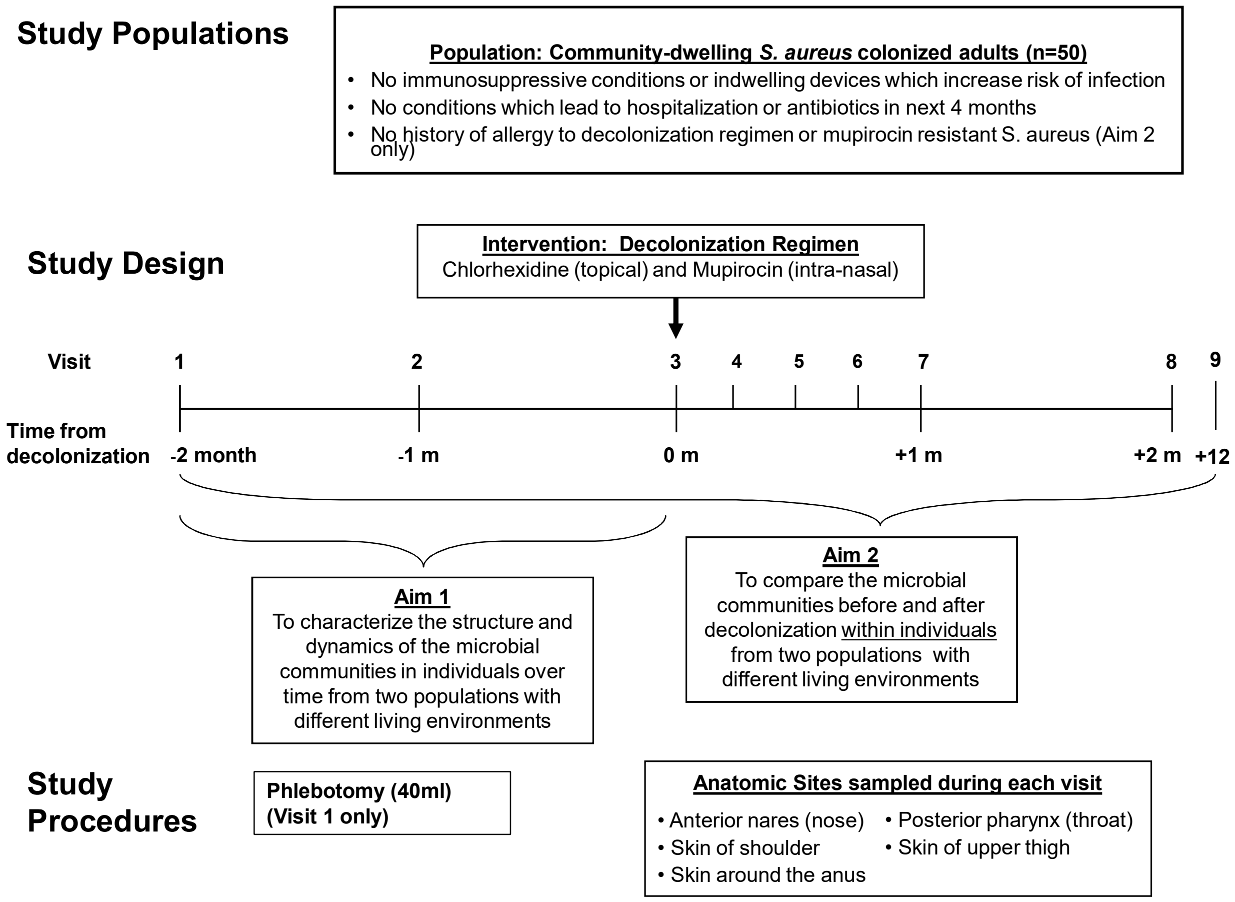

2 Background Information and Rationale

2.1 Background Information

Our adult bodies harbor ~10 times more microbial cells than human cells. Collectively they are known as the human microbiome. A significant number of these microbes are difficult to grow in culture leading to inherent biases in the use of culture based methods to describe it. Our microbiome carries out many metabolic reactions not encoded for in the human genome and are necessary for our health. The NIH funded Human Microbiome Project (HMP) was developed to "to understand the microbial components of the human genetic and metabolic landscape and how they contribute to normal physiology and predisposition to disease" (1). The HMP's focus is on characterizing the microbiome of the gastrointestinal tract, the oropharynx, the anterior nares, the vagina and the skin in young healthy adults.

***Staphylococcus aureus* (SA) is a part of the human microbiome and an important cause of both community and hospital acquired infections.** Methicillin-resistant *S. aureus* (MRSA) is a type of *S. aureus* which is resistant to penicillin-like antibiotics, but susceptible to other antibiotics. *S. aureus* which are not methicillin resistant is referred to as methicillin-susceptible *S. aureus* (MSSA). *S. aureus* is a normal part of the human microbiome. Colonization is frequently prolonged (6) and patients are at risk for infection when the opportunity (e.g. central line or surgery) occurs. Patients with *S. aureus* colonization serve as the source of transmission to others thus preventing transmission decreases infections by reducing the number of patients who serve as a source of transmission. Efforts to decolonize patients with *S. aureus* colonization have not been successful in the long-term (7).

Hospital infection control programs strive to prevent *S. aureus* (and other) infections promoting: hand hygiene, isolation for patients infected or colonized with multi-drug resistant organisms such as MRSA and practices that decrease the risk of specific infections such as maximal barrier precautions during central line placement. Recently forces external to individual hospitals have increased the pressure to prevent infections (8). Medicare will no longer reimburse for certain healthcare associated infections. Many states have made healthcare associated infections publicly reportable (9). In addition, some states (and the national VHA) have legislatively mandated MRSA screening with surveillance cultures (10). This has lead to the growing use of less well established infection control practices including *S. aureus* decolonization.

"Decolonization" is a controversial and rapidly growing strategy to prevent *S. aureus* and other healthcare associated infections. The goal of decolonization is to prevent *S. aureus* infections in the decolonized patients as well as reduce the risk of *S. aureus* transmission to other patients. Decolonization involves the application of targeted or non-targeted antimicrobials to the skin and mucosal surfaces. Two commonly used agents for *S. aureus* decolonization are mupirocin and chlorhexidine.

Mupirocin is an agent with bactericidal activity against Gram-positive bacteria produced by fermentation using the organism *Pseudomonas fluorescens*. It is most commonly applied intra-nasally and is only available by prescription. Two recent meta-analyses concluded that mupirocin decreased *S. aureus* infections in *S. aureus* colonized surgical patients (7, 11).

Intranasal mupirocin is increasingly being used to treat methicillin-resistant *S. aureus* (MRSA) colonization which is detected on mandated surveillance cultures (12). Even more recently a combination of MPC and CHX was shown to decrease surgical site infections due to *S. aureus* (MRSA or MSSA) (13).

Chlorhexidine is a broad spectrum antiseptic agent with activity against Gram-positive and -negative bacteria as well as fungi. It is available as a topical solution ranging in concentration from 0.5% to 4% without a prescription. Chlorhexidine is used in intensive care units (ICU's) to prevent central line associated blood stream infections (14). Chlorhexidine baths have also been shown to decrease MRSA and VRE acquisition in the ICU setting (15). Chlorhexidine was recently shown to be superior to Povidone-Iodine for surgical site antisepsis (16).

2.2 Rationale

Given the role of our normal flora in serving as a barrier to colonization with more pathogenic organisms (aka colonization pressure), decolonization regimens may have unintended negative consequences. For example, preliminary 16S rRNA data suggest that Gram-negative bacilli from the orders Enterobacteriales and Pseudomonadales are present as a component of the human microbiome of the anterior nares. Decolonization with intranasal mupirocin, a Gram-positive antimicrobial agent, may increase this Gram-negative colonization and consequently increase the likelihood of infections due to these pathogens. The recent Cochrane Review of Mupirocin ointment for Preventing *S. aureus* infections in nasal carriers concluded that the use of mupirocin led to a relative increase in infections due to bacteria other than *S. aureus* (Analysis 6.1: RR 1.4 95%CI 1.1-1.7) and that infections due to Gram-negative bacilli were increased but not statistically significant (Analysis 6.3: RR 1.7 95%CI 0.8-3.5)(7). Although suggestive, these analyses are limited in scope because few of the mupirocin clinical trials have reported overall infections. Many, including the recent NEJM paper, have just focused on *S. aureus* infections (13). Furthermore these trials have not looked at the impact of mupirocin on colonization with other organisms. If mupirocin promotes colonization with Gram-negative bacilli, this is an important adverse event given that colonization with Gram-negative bacilli increases the risk of Gram-negative bacilli infection in hospitalized patients (17-19) and the increasing antibiotic resistance in the Enterobacteriaceae, Pseudomonas and Acinetobacter over the past decade (20). Our short term goal is to determine if these increasingly used decolonization regimens, targeted at controlling MRSA in particular, may result in a secondary negative effect of promoting colonization with pathogenic Gram-negative bacilli. This could be an unintended consequence of healthcare policies which are indirectly promoting the use of these decolonization regimens and would have an impact of the use of mupirocin for infection control purposes. The long term goal of this research is to use the information gained to develop new ways to manipulate the human microbiome to reduce the risk of *S. aureus* infections with minimized negative consequences.

2.3 Risk/Benefits

The risks of the proposed study are minimal. There are possible risks and discomforts from this study for participants.

BACTROBAN NASAL (mupirocin calcium ointment, 2%) is a licensed, widely used, and generally well tolerated topical antibiotic available by prescription. Reported side effects noted in the packet insert include: Headache, 9%; Rhinitis, 6%; Respiratory disorder, including

upper respiratory tract congestion, 5%; Pharyngitis, 4%; Taste perversion, 3%; Burning/stinging, 2% ; Cough, 2% and Pruritus, 1%.

Antimicrobial antiseptic skin cleanser (4% chlorhexidine), is an FDA- approved formulation and application for topical 4% chlorhexidine, an antiseptic solution which is available without a prescription. It is widely used for pre-operative skin cleansing and extremely well tolerated. Mild skin irritation can occur.

The sampling methods for the anterior nares, throat, skin, and perirectal skin sites is physically non-invasive, but there could some discomfort during the procedure. The potential discomfort from obtaining the specimens does not place any participant at physical risk. While taking the throat culture, the participant may gag and possibly vomit. A trained research coordinator will use very gentle pressure while performing the cultures to minimize these risks. However, there is the possibility of psychological discomfort from the specimen collection.

The drawing of blood may cause brief discomfort or slight pain and, rarely, fainting. Bruising at the site of the blood draw may occur but can be prevented or lessened by applying pressure for several minutes. Infection at the site where blood is drawn is not likely, and this risk will be minimized by the use of alcohol swabs and sterile gauze.

There is a risk that health information could be in accidentally disclosed to others outside the study. Confidentiality will be protected to the fullest extent permitted by law. Despite the recent passage of a federal law that bars genetic discrimination in employment and some types of insurance, the use by others of genetic information from the eventual study of the blood specimens could conceivably impact the participant negatively in some way. For example, there is a very small risk that genetic information obtained by this study could be used by law enforcement officials to try to learn more about the participants or their family members for the purpose of a criminal investigation.

There may be adverse events that are not yet known. For example, we are studying whether mupirocin leads to an increase in Gram-negative colonization in the anterior nares and throat which might lead to an increased risk of Gram-negative infections. To date, there is evidence that mupirocin leads to an increase in the relative risk of infections due to organisms other than *S. aureus* including Gram-negative bacilli (7); however, this was in surgical and dialysis patients, a group at increased risk for infections in genera and was not associated with an absolute risk of infectionl. In general, Gram-negative bacilli are opportunistic pathogens. They colonize frequently, but infrequently cause infection. To minimize the risk of infection, we are excluding older adults who would be at increased risk of infection (e.g. surgical and dialysis patients, those with recent hospitalizations, history of urinary tract infections).

There is no alternative treatment or procedure to participating in this study, but individuals have the right to decline participation in this research without any risk.

There are no direct benefits to study participants. Participants will receive a regimen that will eradicate *S. aureus* colonization in the short term. Although *S. aureus* colonization contributes to the risk of *S. aureus* infection, the absolute risk of *S. aureus* infection is small enough that this is not believed to be a direct benefit. This study will contribute knowledge about the human microbiome. *S. aureus* and other opportunistic pathogens are members of this

microbiome. When the opportunity arises (e.g. a person has surgery or a central venous catheter), these bacteria can cause an infection particularly in hospitalized patients. Understanding the human microbiome in *S. aureus* colonized participants can help us develop new ways to prevent *S. aureus* infections.

2.4 Study Conduct

This study will be conducted in compliance with the protocol approved by the UMB Institutional Review Board (hereafter referred to as IRB) and VAMHCS R&D Committee and according to Good Clinical Practice standards and the VHA Handbook 1200.05 Requirements for the Protection of Human Subjects in Research. No deviation from the protocol will be knowingly implemented without the prior review and approval of the IRB except where it may be necessary to eliminate an immediate hazard to a research participant. In such case, the deviation will be reported to the IRB as soon as possible.

3 Study Objectives

This is a mechanistic patient-oriented research study designed to characterize the baseline microbiome of body sites associated with *S. aureus* colonization and to understand the effect of a local decolonization regimen on the microbiome of the nose, throat and three skin sites in *S. aureus* colonized adults.

There are two additional objectives. The first is to determine the immune responses to organisms that are identified in the microbiome. Specifically, this objective will assess whether participants with successful decolonization of *S. aureus* colonization have higher *S. aureus* specific antibody levels than participants without successful decolonization of *S. aureus* colonization. Other responses to other bacteria may be looked at in future studies. The second is to examine the relationship of host genotype (by either genotyping or sequencing the DNA) to the microbiota present on/in an individual. Specifically, this objective will assess whether participants with successful eradication of *S. aureus* colonization have a different genotype than participants without successful eradication of *S. aureus* colonization.

4 Study Design

4.1 Primary Study Endpoints/Secondary Endpoints

When examining the baseline microbiome as a whole, characterizing the distribution of 16S bacterial sequences present is the ‘endpoint’. The study will also look at changes to the baseline microbiome following decolonization regimens.

4.2 Study Design/Type

This is a single-center, interventional study to describe the microbiome sequentially over a 16 week period in *S. aureus* colonized adults, both before and after administration of topical antiseptic agent (CHLORHEXIDINE) and intranasal antimicrobial ointment (MUPIROCIN). Eighty male and female community dwelling participants residing in the Baltimore area will be recruited and screened to document their health status, with the goal of 50 participants completing the study.

4.3 Duration

Study participants will have a total of 9 cultures of the anterior nares, posterior pharynx, skin over the subclavian vein, skin over the femoral vein and perirectal skin over a period of approximately 52 weeks.

5 Selection and Withdrawal of Participants

5.1 Recruitment of Study population

All study participants will be adults that live in Baltimore or the surrounding area. Community dwelling participants will be recruited from the Managed Care Clinics of the Baltimore VA Medical Center (via an IRB approved flier), by conducting a screening of the VA medical records for patients with cultures positive for S. aureus in the last three years in the VA Maryland HCS (via an IRB approved letter mailing), and approved VA research registries (via an IRB approved letter). In addition, we will recruit from students and employees of the University of Maryland Baltimore and the VA Maryland HCS (via an IRB approved flier).

When contacted about participation, the study coordinator will review the information from the study flier or letter with the potential participant and answer questions. If the potential subject is interested in participating, the study coordinator will review the eligibility criteria prior to scheduling the Enrollment Visit in the General Clinical Research Center (GCRC) in order to screen out ineligible participants by history. Identifying information will only be collected on those potential participants who are scheduled for the screening/enrollment visit.

5.2 Inclusion Criteria

- Adults living independently in the Baltimore area
- Willing and able to provide anterior nares, throat, skin, and perirectal specimens over up to an 18 week time period.
- Willing and able to administer intranasal mupirocin and topical chlorhexidine over a five day period.
- Capable of understanding and complying with the entire study protocol.
- Provided signed and dated informed consent.
- *S. aureus* colonization on at least one of the enrollment cultures (after informed consent)

5.3 Exclusion Criteria

- Use of anticancer chemotherapy or radiation therapy (cytotoxic) within the past 6 months
- History of HIV infection with most recent CD4 of <200
- Immunosuppression medications within the past 3 months
- Use of systemic antibacterial or antifungal agents in the past 3 months
- Use of nasal steroids currently or in the past 3 months
- Use of nasal antimicrobial ointment in the past 3 months
- Any current indwelling percutaneous medical device or urinary catheter
- Acute care hospitalization in the past 3 months
- Planned surgery or hospitalization during the study period

- History of an allergic reaction to chlorhexidine or mupirocin
- Oral temperature of >100 F at enrollment visit
- BMI <18 or >35 at enrollment visit

5.4 Participant Withdrawal

Study participants will be withdrawn from the study if found to have been initially ineligible or incapable of following through with mupirocin or chlorhexidine treatment regimen. Study participants will be withdrawn if all of their specimens from visit 1 are negative for *S. aureus* by microbiological culture. These participants will be contacted by phone. No further follow-up with withdrawn participants is required.

5.5 Medication

All medications are permitted during the study period. It is possible that some study participants will take antibiotics for clinical reasons during their time in the study. They will continue to be followed throughout the study as a 'natural' experiment, but their post-antibiotic data will be excluded from the primary analysis.

6 Informed Consent Process for Participants

At the initial visit, the study coordinator will verbally review the written informed consent and HIPAA form with the potential participant in a private area. The potential participant will be asked if he/she wishes for the study coordinator to read the consent form verbatim or to summarize it as he/she follows along.

Because cognitive impairment is possible in the adult population and participants need to be "Capable of understanding and complying with the entire study protocol", each potential participant will be evaluated for his/her ability to give informed consent. The potential participant must be alert and able to communicate in order to give informed consent. He /she will be asked the following questions before informed consent to participate is considered to have occurred: "Why are we doing the study? Does the study have any risks? What are you going to do in the study? Do you have to participate in the study? If you don't want to be in the study anymore, what do you do?" as described in <u>Evaluation to Sign Consent</u>. If the potential participant is unable to answer these questions correctly after two opportunities, he/she will not be eligible.

7 Study Intervention

7.1 Study Product Description

7.1.1 Acquisition

BACTROBAN NASAL ointment (mupirocin calcium ointment, 2%) and antimicrobial antiseptic skin cleanser (4% chlorhexidine) will be shipped to the VAMHCS Investigational Pharmacy.

7.1.2 Formulation, Packaging, and Labeling

The study products are BACTROBAN NASAL ointment (mupirocin calcium ointment, 2%) and antimicrobial antiseptic skin cleanser (4% chlorhexidine). BACTROBAN NASAL is a licensed medication packaged and labeled by GlaxoSmithKline. BACTROBAN NASAL (mupirocin calcium ointment, 2%) is packaged in 1.0-gram tubes and is for intranasal use only. Antimicrobial antiseptic skin cleanser (4% chlorhexidine) is an FDA-approved medication packaged in 16 oz containers.

7.1.3 Product Storage and Stability

BACTROBAN NASAL ointment (mupirocin calcium ointment, 2%) and antimicrobial antiseptic skin cleanser (4% chlorhexidine) will be stored between 20-25°C (68-77°F), as directed by the manufacturers.

7.2 Dosage, Preparation and Administration of Study Intervention

Participants will be self-treated with antimicrobial antiseptic skin cleanser (4% chlorhexidine) topically 3 times within 5 days before the Week 9 visit. Participants will also be self-treated with BACTROBAN NASAL ointment (mupirocin calcium ointment, 2%) intranasally in the 5 days before the Week 9 visit.

7.3 Modification of Study Intervention

If a participant develops allergic symptoms or other signs of intolerance to antimicrobial antiseptic skin cleanser (4% chlorhexidine) or BACTROBAN NASAL, that medication will be stopped.

7.4 Accountability Procedures for the Study Intervention

The PI is responsible for ensuring that a current record of product disposition is maintained and product is dispensed only at an official study site by authorized personnel as required by applicable regulations and guidelines.

Antimicrobial antiseptic skin cleanser (4% chlorhexidine) and BACTROBAN NASAL ointment (mupirocin calcium ointment, 2%) will be distributed to participants by the General Clinical Research Center at the University of Maryland Medical Center. Documentation of all products received, distributed and destroyed will be kept by the research pharmacist. An assigned study monitor will review the pharmacy records for accuracy and completeness.

7.5 Assessment of Participant Compliance with Study Intervention

At the Week 8 study visit, three doses of antimicrobial antiseptic skin cleanser (4% chlorhexidine) and ten doses of BACTROBAN NASAL ointment (mupirocin calcium ointment, 2%) will be dispensed to all participants. Instructions regarding dosing, missed doses and possible side effects will be provided. In addition, a Compliance log will be provided for the participant to document the date and time required study doses were taken. In addition, missed doses will be recorded on this log and the participant will be instructed to call the Investigator if two or more doses are missed. The Investigator may decide to provide the participant with an additional two doses.

At the Week 9 study visit, the log will be reviewed to determine compliance with study medication.

8 Study Procedures/Evaluations

8.1 Clinical Evaluations

Day 0 (enrollment visit) – Will occur in the GCRC:

- Screen participant regarding inclusion and exclusion criteria.
- Obtain informed consent.
- Obtain demographic information and a brief medical history by interview. The brief medical history includes current and recent medications and past medical and surgical history.
- Collect vital signs and basic anthropometric measurements in order to determine eligibility. Participants deemed eligible for the study will be enrolled at this visit.
- Measure oral temperature to screen out participants with febrile infectious disease.
- Complete a brief physical assessment of the culture sites.
- Obtain a 40 mL blood sample and specimens from the following sites: anterior nares, posterior pharynx, skin over the femoral vein, skin over the subclavian vein, and perirectal skin (see SOP for Methods).
- Counsel participant to refrain from using any antibacterial/antiseptic products for 48 hours before visits.
- Counsel participant to refrain from swimming in a chlorinated pool or using a hot tub in the 48 hours before visits.
- Counsel participant to refrain from bathing or showering in the 12 hours prior to the study visit.
- Inform participant that if specimens are negative for *S. aureus*, he/she will be withdrawn from the study at week 4.

For participants who do NOT meet eligibility criteria to continue in the study: (if MRSA and MSSA negative on all enrollment cultures)

1) Inform participant via phone that all enrollment specimens were MRSA and MSSA negative and withdraw him/her from the study.
2) Complete Participant Deactivation Form

Week 4 – Will occur in the GCRC:

- Review results of cultures from Day 0 to determine eligibility
- Reconfirm participant's willingness to participate in the study.
- Confirm that participant has abstained for the last 48 hours from the following: using any antibacterial/antiseptic products, swimming in a chlorinated pool or using a hot tub.
- Record time and date of last bath or shower.
- Measure oral temperature to screen out participants with febrile infectious disease.
- Collect a focused medical history.
- Review concomitant medications

- Complete a brief physical assessment of their anterior nares, posterior pharynx and skin sites.
- Obtain specimens from the anterior nares, posterior pharynx, skin over subclavian vein, skin over femoral vein and perirectal skin.
- Counsel participant to refrain from using any antibacterial/antiseptic products for 48 hours before visits.
- Counsel participant to refrain from swimming in a chlorinated pool or using a hot tub in the 48 hours before visits.
- Counsel participant to refrain from bathing or showering in the 12 hours prior to the next study visit.

<u>Week 8 – Will occur in the GCRC:</u>

- Reconfirm participant's willingness to participate in the study.
- Confirm that participant has abstained for the last 48 hours from the following: using any antibacterial/antiseptic products, swimming in a chlorinated pool or using a hot tub.
- Record time and date of last bath or shower.
- Measure oral temperature to screen out participants with febrile infectious disease.
- Collect a focused medical history.
- Review concomitant medications
- Complete a brief physical assessment of their anterior nares, posterior pharynx and skin sites.
- Obtain specimens from the anterior nares, posterior pharynx, skin over subclavian vein, skin over femoral vein and perirectal skin.

For participants who meet eligibility criteria to continue in the study:

- Provide a 16oz of antimicrobial antiseptic skin cleanser (4% chlorhexidine) to participant. Educate participant on how to use the skin cleanser in the shower on all of their skin from the neck down.
- Provide ten doses of mupirocin ointment to participants. Educate participant on how to self-apply the ointment intranasally.
- For each medication, provide instructions regarding application, dosing, missed doses and possible side effects.
- Provide a Compliance Log to participant to document the date and time required study doses are taken. Instruct participant to also record missed doses on the log, and to call the Investigator if two or more doses are missing. The Investigator may decide to provide the participant with an additional two doses.
- Counsel participant to refrain from using any antibacterial/antiseptic products for 48 hours before visits.
- Counsel participant to refrain from swimming in a chlorinated pool or using a hot tub in the 48 hours before visits.
- Counsel participant to refrain from bathing or showering in the 12 hours prior to the next study visit.

<u>Week 9 – Will occur in the GCRC:</u>

- Reconfirm participant's willingness to participate in the study.
- Confirm that participant has abstained for the last 48 hours from the following: using any antibacterial/antiseptic products, swimming in a chlorinated pool or using a hot tub.
- Record time and date of last bath or shower.
- Measure oral temperature to screen out participants with febrile infectious disease.
- Collect a focused medical history.
- Review concomitant medications.
- Complete a brief physical assessment of their anterior nares, posterior pharynx and skin sites.
- Obtain specimens from the anterior nares, posterior pharynx, skin over subclavian vein, skin over femoral vein and perirectal skin.
- Review the Compliance Log to determine compliance with the 5-day course of mupirocin/chlorhexidine.
- Counsel participant to refrain from using any antibacterial/antiseptic products for 48 hours before visits.
- Counsel participant to refrain from swimming in a chlorinated pool or using a hot tub in the 48 hours before visits.
- Counsel participant to refrain from bathing or showering in the 12 hours prior to the next study visit.

Weeks 10, 11, 12, and 16– Will occur in the GCRC:

- Reconfirm participant's willingness to participate in the study.
- Confirm that participant has abstained for the last 48 hours from the following: using any antibacterial/antiseptic products, swimming in a chlorinated pool or using a hot tub.
- Record time and date of last bath or shower.
- Measure oral temperature to screen out participants with febrile infectious disease.
- Collect a focused medical history.
- Review concomitant medications
- Complete a brief physical assessment of their anterior nares, posterior pharynx and skin sites.
- Obtain specimens from the anterior nares, posterior pharynx, skin over subclavian vein, skin over femoral vein and perirectal skin.
- Counsel participant to refrain from using any antibacterial/antiseptic products for 48 hours before visits.
- Counsel participant to refrain from swimming in a chlorinated pool or using a hot tub in the 48 hours before visits.
- Counsel participant to refrain from bathing or showering in the 12 hours prior to the next study visit.

Month 12 (+/- 3 months) Follow-up visit- Will occur in the GCRC

Clinical procedures are completed by GCRC staff. However, in extreme circumstances it is possible for this study visit to be completed at the participant's home, in which case the follow-up visit procedures are completed by a trained research staff member.

- Participants previously enrolled prior to 12 month follow-up visit being added, will need to be re-consented at the 12 month follow-up visit prior to any data/specimen collection.
- Participants who were consented for the 12 month follow-up visit will have the consent form reviewed with them prior to any data/specimen collection.

1) Obtain nose culture – see Appendix 2 "Method for Obtaining Nose Culture".
2) Obtain throat culture – see Appendix 3 "Method for Obtaining Throat Culture".
3) Obtain skin cultures – see Appendix 4 "Method for Obtaining Skin Cultures".
4) Interview participant and complete the follow up visit form.

The visit time points represent ideal visit times, but this exact timing is not required. We will be using the following guidelines:

1) All follow-up visits through visit 8 must occur no more than 18 weeks after enrollment.
2) Participants should have 3 sets of specimens collected in the 2 months prior to administration of chlorhexidine/mupirocin. These initial 3 sets of specimens can occur no closer than 2 weeks apart.
3) Participants should have 4 sets of specimens collected in the 4 weeks after chlorhexidine/mupirocin administration. These 4 sets of specimens can occur no closer than 5 days apart.
4) Participants should have an 8thset of specimens collected after the 4 weekly sets of specimens. This 8th set of specimens can occur no closer than 2 weeks after the last weekly specimens (which should have been collected at approximately week 12).
5) The 12 month follow-up visit should occur between the 9th and the 15th month after the partciapants original date of enrollment in the study.

Specimens not collected at the designated time or missed altogether will not be reported as deviations. Missed visits will not be reported as deviations.

See SOPS for detailed procedures of visit conduct, specimen collection, and case report forms.

8.2 Laboratory Evaluations

During each study visit a specimen is obtained by trained research personnel from each of the following sites: anterior nares, posterior pharynx, skin over the femoral vein, skin over the subclavian vein, and perirectal skin. These specimens are tested for MRSA, MSSA and gram-negative bacilli in the Research Microbiology Laboratory and are also analyzed for the presence of all bacteria using metagenomics techniques.

In addition, a blood sample taken at the enrollment visit will be initially processed in the GCRC and transported to the Research Microbiology Lab for storage until immunologic and genotypic tests are performed.

9 Unanticipated Problems and Serious Adverse Events

The methods of specimen collection in this microbiome sampling study pose only minimal risk to the study participants. As defined in 45 US Code of Federal Regulations (CFR) 6.102 (i), "Minimal risk means that the probability and magnitude of harm or discomfort anticipated in the research are not greater in and of themselves than those ordinarily encountered in daily life or during the performance of routine physical or psychological examinations or tests." The minimal

physical risks associated with the sampling procedures are described in Section 1.3 and in the informed consent document.

The nature of the information collected from participants may involve risk to their privacy. These risks are described in Section 2.3 and in the informed consent document.

The results of research cannot be foreseen, so it is possible that unanticipated problems may arise in the study. In addition to unexpected adverse events, there are other types of incidents, experiences, and outcomes that occur during the conduct of human subjects research that represent unanticipated problems but are not considered adverse events. For example, some unanticipated problems involve social or economic harm instead of the physical or psychological harm associated with adverse events. The investigator or designee is responsible for the detection and documentation of unanticipated problems and Serious Adverse Events (SAE) in participants participating in the study. Unanticipated problems and SAEs should be reported to the IRB and the VAMHCS PO and ISO per VA handbook 6500 and as outlined in the Manual of Procedures.

We will use the definition of Unanticipated Problems as specified in the VHA Handbook 1058.01. We will follow the reporting requirements specified in the VHA Handbook 1058.01. We will collect all of the information required to follow those reporting requirements.

10 Quality Control and Quality Assurance

The PI and affiliated institution are responsible for conducting the study in compliance with the IRB-approved protocol, all applicable federal and state regulations, VHA Handbook 1200.05 Requirements for the Protection of Human Subjects in Research and the International Conference on Harmonization, E6 Good Clinical Practice by adhering to the requirements for collecting, documenting and reporting complete and accurate data. A separate Data Quality Assurance (QA) Plan will be developed for this protocol to describe the frequency of review of study related data and the roles and responsibilities for activities related to Quality Control (QC).

11 Statistical Plan

11.1 Analysis Plan for Microbial Community Profiling

In this project, we will utilize model-based statistical methods to study the dynamics and composition of the microbial communities present in the anterior nares, posterior pharynx and three skin sites in two populations (Protocol HP-51033 Nursing home dwelling MRSA Colonized Adults and this protocol) with and without current exposure to the healthcare environment, and before and after decolonization using treatment with topical chlorhexidine and intranasal mupirocin. These analyses will be performed in association with the various metadata collected and will be critical to be able to answer the various questions associated with our project, which include:

Aim 1:
Over time within individuals and at the nose and throat, and skin sites- what are the microbial communities present, their relative/absolute abundance?
Are there differences between individuals at the nose and throat by living environment (community-dwelling vs. nursing home dwelling)?
Aim 2:

What are the changes, within individuals, in the microbiome associated with decolonization over time at the nose, throat, and skin sites in term or composition and relative/absolute abundance?
What are the changes in the microbiome of each individual from the baseline to after decolonization at the nose, throat, and skin sites, in relation to decolonization and living environment (community-dwelling vs. nursing home dwelling)?

The assignment of 16S sequencing reads to OTUs will be performed using OTUPicker (23, 24), the Ribosomal Database Project Bayesian classifier (25) and Hidden Markov Chain models-based algorithms developed at UMD-IGS by Drs. Jacques Ravel and Pawel Gajer to classify a subgroup of OTUs to the species level (for details about the speciation analysis, see (26)). OTU classification data for each sample will then be utilized for model-based comparative analyses of community structure. Model-based approaches directly use the observed raw data, and minimize the loss of information by eliminating the data reduction steps associated with distance-based methods (used in OTU classification tools such as DOTUR (24), UNIFRAC (25) and SONS (23)). The Bayesian and likelihood statistical frameworks used in these approaches provide a probabilistic measure of confidence in the results and facilitate comparisons between the different models and methods through the use of model-selection strategies such as Bayes factors, Akaike's information criterion, Bayesian information criterion and decision theory, and hypothesis-testing approaches. Moreover, model-based approaches allow for incorporation of metadata along with data used to infer the group membership of OTUs that comprise the microbial communities under study. This information is then used to cluster these communities. Model-based approaches also provide a classification tool to assign newly sampled individuals to pre-existing groups that result from clustering approaches that take both metadata and microbial community composition into account.

11.2 Analysis Plan for Microbiologic Data

The goals of the microbiological analysis are two-fold. The first goal is descriptive to document the effectiveness of the regimen in reducing colonization with MRSA, MSSA and pathogenic Gram-negative bacilli at the body sites sampled. The second goal will compare the effectiveness of the regimen in the two different populations. We will assess whether there are differences in acquisition of GNB and antibiotic resistant GNB between community-dwelling and nursing home-dwelling adults. We will compare weeks to MRSA or MSSA re-colonization in each body site by living environment using survival curves and the Wilcoxon log rank test. We will compare whether there are differences in the acquisition (among those negative at baseline) of specific GNB and resistant GNB between community-dwelling and nursing home-dwelling, MRSA or MSSA-colonized adults using chi-square tests or Fisher's exact test as appropriate.

11.3 Sample Size Estimates

How many participants (or specimens, or charts) will be used in this study?
Local: 250   Worldwide: 250

We plan on enrolling 250 participants to end up with a final sample size of 40. We assume that approximately one in five participants will have *S. aureus* colonization at the screening/enrollment visit. We assume that 10 participants will become ineligible during the course of the protocol because of hospitalization or antibiotic administration. The final sample

size was chosen based on feasibility and cost. Unlike more traditional genetic association studies, where theoretical sample calculations can be performed, the current study uses more novel data mining approaches applied to data obtained from high-throughput genotyping technology, in which many different potential OTUs are compared between groups (pre and post decolonization within a participant). Currently, there is no generally accepted theoretical power calculations for the analyses proposed here. Since the participants will serve as their own controls (pre and post decolonization), we increase power to detect significant differences and potentially avoiding confounding factors (27).

12 Ethical Considerations

This study will be conducted according to US and international standards of Good Clinical Practice (FDA regulations 21 CFR 312 for IND studies and FDA guidance E6) for all studies. Applicable government regulations, VHA Handbook 1200.05 Requirements for the Protection of Human Subjects in Research and UMB research policies and procedures will also be followed.

All participants for this study will be provided a consent form describing this study and providing sufficient information for participants to make an informed decision about their participation in this study. This consent form will be submitted with the protocol for review and approval by the IRB. The formal consent of a participant, using the IRB-approved consent form, will be obtained before that participant is submitted to any study procedure. This consent form must be signed by the participant, and the investigator-designated research professional obtaining the consent.

13 Data Handling and Record Keeping

The Investigator is responsible to ensure the accuracy, completeness, legibility, and timeliness of the data reported. All source documents will be completed in a legible manner to ensure accurate interpretation of data. Black ink is required to ensure clarity of reproduced copies. When making changes or corrections, the original entry will be crossed out with a single line, and the change initialed and dated. Erasing, overwriting, or use of correction fluid or tape will not be done.

All source documents and laboratory reports will be reviewed by the clinical team and data entry staff, who will ensure that they are accurate and complete. AEs must be graded, assessed for severity and causality, and reviewed by the site PI or designee. Data collection is the responsibility of the study staff. During the study, the Investigator must maintain complete and accurate documentation for the study.

Copies of the CRFs will serve as source documents and maintained for recording data for each participant enrolled in the study. Research records generated in this study will be stored in file cabinets in a locked room and on a secure electronic database. Only authorized personnel will have access to the data. Access will be revoked when study (research) staff are no longer part of the study, and/or at conclusion of the study.

All data will be maintained for the period of time stated in the applicable Privacy Act System of Records notice, Records Control Schedule (RCS) 10-1, and VA policy. Identifiable information will not be destroyed except with appropriate destruction authority. The research data will be destroyed in accordance with current RCS 10-1 requirements. At this time, there is a moratorium on all research data.

Cultures before and after Decolonization in Community Dwelling Adults with Current *S. aureus* Colonization

14 References

1. Turnbaugh PJ, Ley RE, Hamady M, Fraser-Liggett CM, Knight R, Gordon JI. The human microbiome project. *Nature.* 2007; 449(7164): 804-810.

6. Kluytmans J, van Belkum A, Verbrugh H. Nasal carriage of staphylococcus aureus: Epidemiology, underlying mechanisms, and associated risks. *Clin Microbiol Rev.* 1997; 10(3): 505-520.

7. van Rijen M, Bonten M, Wenzel R, Kluytmans J. Mupirocin ointment for preventing staphylococcus aureus infections in nasal carriers. *Cochrane Database Syst Rev.* 2008(4): CD006216.

8. Arias KM. Mandatory reporting and pay for performance: Health care infections in the limelight. *AORN J.* 2008; 87(4): 750-758.

9. Meier BM, Stone PW, Gebbie KM. Public health law for the collection and reporting of health care-associated infections. *Am J Infect Control.* 2008; 36(8): 537-551.

10. Weber SG, Huang SS, Oriola S, Huskins WC, Noskin GA, Harriman K, Olmsted RN, Bonten M, Lundstrom T, Climo MW, Roghmann MC, Murphy CL, Karchmer TB, Society for Healthcare Epidemiology of America, Association of Professionals in Infection Control and Epidemiology. Legislative mandates for use of active surveillance cultures to screen for methicillin-resistant staphylococcus aureus and vancomycin-resistant enterococci: Position statement from the joint SHEA and APIC task force. *Infect Control Hosp Epidemiol.* 2007 Mar; 28(3): 249-260.

11. Ammerlaan HS, Kluytmans JA, Wertheim HF, Nouwen JL, Bonten MJ. Eradication of methicillin-resistant staphylococcus aureus carriage: A systematic review. *Clin Infect Dis.* 2009; 48(7): 922-930.

12. Robicsek A, Beaumont JL, Paule SM, Hacek DM, Thomson RB,Jr., Kaul KL, King P, Peterson LR. Universal surveillance for methicillin-resistant staphylococcus aureus in 3 affiliated hospitals. *Ann Intern Med.* 2008; 148(6): 409-418.

13. Bode LG, Kluytmans JA, Wertheim HF, Bogaers D, Vandenbroucke-Grauls CM, Roosendaal R, Troelstra A, Box AT, Voss A, van der Tweel I, van Belkum A, Verbrugh HA, Vos MC. Preventing surgical-site infections in nasal carriers of staphylococcus aureus. *N Engl J Med.* 2010 Jan 7; 362(1): 9-17.

14. Bleasdale SC, Trick WE, Gonzalez IM, Lyles RD, Hayden MK, Weinstein RA. Effectiveness of chlorhexidine bathing to reduce catheter-associated bloodstream infections in medical intensive care unit patients. *Arch Intern Med.* 2007; 167(19): 2073-2079.

15. Climo MW, Sepkowitz KA, Zuccotti G, Fraser VJ, Warren DK, Perl TM, Speck K, Jernigan JA, Robles JR, Wong ES. The effect of daily bathing with chlorhexidine on the acquisition of

methicillin-resistant staphylococcus aureus, vancomycin-resistant enterococcus, and healthcare-associated bloodstream infections: Results of a quasi-experimental multicenter trial. *Crit Care Med.* 2009; 37(6): 1858-1865.

16. Darouiche RO, Wall MJ,Jr, Itani KM, Otterson MF, Webb AL, Carrick MM, Miller HJ, Awad SS, Crosby CT, Mosier MC, Alsharif A, Berger DH. Chlorhexidine-alcohol versus povidone-iodine for surgical-site antisepsis. *N Engl J Med.* 2010 Jan 7; 362(1): 18-26.

17. Harris AD, McGregor JC, Johnson JA, Strauss SM, Moore AC, Standiford HC, Hebden JN, Morris JG,Jr. Risk factors for colonization with extended-spectrum beta-lactamase-producing bacteria and intensive care unit admission. *Emerging Infect Dis.* 2007; 13(8): 1144-1149.

18. Thom KA, Johnson JA, Strauss SM, Furuno JP, Perencevich EN, Harris AD. Increasing prevalence of gastrointestinal colonization with ceftazidime-resistant gram-negative bacteria among intensive care unit patients. *Infect Control Hosp Epidemiol.* 2007; 28(11): 1240-1246.

19. Selden R, Lee S, Wang WL, Bennett JV, Eickhoff TC. Nosocomial klebsiella infections: Intestinal colonization as a reservoir. *Ann Intern Med.* 1971 May; 74(5): 657-664.

20. Hidron AI, Edwards JR, Patel J, Horan TC, Sievert DM, Pollock DA, Fridkin SK. NHSN annual update: Antimicrobial-resistant pathogens associated with healthcare-associated infections: Annual summary of data reported to the national healthcare safety network at the centers for disease control and prevention, 2006-2007. *Infect Control Hosp Epidemiol.* 2008; 29(11): 996-1011.

21. Harbarth S, Dharan S, Liassine N, Herrault P, Auckenthaler R, Pittet D. Randomized, placebo-controlled, double-blind trial to evaluate the efficacy of mupirocin for eradicating carriage of methicillin-resistant staphylococcus aureus. *Antimicrob Agents Chemother.* 1999; 43(6): 1412-146.

22. CLSI. Performance standards for antimicrobial susceptibility testing; twenty-first informational supplement. Wayne, PA: Clinical and Laboratory Standards Institute; 2011.

23. Schloss PD, Handelsman J. Introducing SONS, a tool for operational taxonomic unit-based comparisons of microbial community memberships and structures. *Appl Environ Microbiol.* 2006 Oct; 72(10): 6773-6779. PMCID: PMC1610290.

24. Schloss PD, Handelsman J. Introducing DOTUR, a computer program for defining operational taxonomic units and estimating species richness. *Appl Environ Microbiol.* 2005 Mar; 71(3): 1501-1506. PMCID: PMC1065144.

25. Lozupone C, Knight R. UniFrac: A new phylogenetic method for comparing microbial communities. *Appl Environ Microbiol.* 2005 Dec; 71(12): 8228-8235. PMCID: PMC1317376.

26. Ravel J, Gajer P, Abdo Z, Schneider GM, Koenig SS, McCulle SL, Karlebach S, Gorle R, Russell J, Tacket CO, Brotman RM, Davis CC, Ault K, Peralta L, Forney LJ. Microbes and

health sackler colloquium: Vaginal microbiome of reproductive-age women. *Proc Natl Acad Sci U S A.* 2010 Jun 3.

27. Klevens RM, Edwards JR, Richards CL,Jr., Horan TC, Gaynes RP, Pollock DA, Cardo DM. Estimating health care-associated infections and deaths in U.S. hospitals, 2002. *Public Health Rep.* 2007; 122(2): 160-166.
