# Supplementary material for: Effect of mupirocin for Staphylococcus aureus decolonization on the microbiome of the nose and throat in community and nursing home dwelling adults
Source: PLoS One. 2021 Jun 8;16(6):e0252004. doi: 10.1371/journal.pone.0252004 (PMC8186807; doi:10.1371/journal.pone.0252004)
Supplement: S2 File — (PDF) [file pone.0252004.s010.pdf]

PROTOCOL NAME
Human Microbiome and Health Care Associated Infection – Nursing Home Dwelling Older Veterans

Grant: Human Microbiome and Health Care Associated Infection (1 R01 AI087865)

PROTOCOL IDENTIFYING NUMBER (any amendments should bear the amendment number)

CICERO protocol number: HP-58923

PROTOCOL VERSION
Modification 5
DATE: 1/26/2015

Commonly Used Abbreviations

MRSA	Methicillin-resistant *S. aureus*
VAMHCS R&D	VA Maryland Health Care System Research and Development
UMB IRB	University of Maryland Baltimore Institutional Review Board

GENERAL INFORMATION

Name and address of the study monitor and person authorized to sign the protocol and amendments:

Mary-Claire Roghmann, MD, MS
University of Maryland School of Medicine
Department of Epidemiology and Public Health
10 South Pine Street, MSTF 336, 360
Baltimore, MD 21201

Name and addresses of the clinical laboratories and/or other institutions involved in the study:

Laboratories-

Baltimore VA Medical Center
Microbiology Research Lab, 3B-112
10 North Greene Street
Baltimore, MD 21201

Microbiology Research Lab
Department of Pathology
Allied Health Building, Room 401
100 Penn Street
Baltimore, MD 21201

Institute for Genome Sciences (IGS)
University of Maryland School of Medicine
801 W Baltimore St
BioPark II, 6th Floor
Baltimore, MD 21201

Clinical Sites-

Perry Point VA Medical Center
Community Living Center
Perry Point, MD 21902

Loch Raven VA Community Living &
Rehabilitation Center
3900 Loch Raven Boulevard
Baltimore, MD 21218

Table of Contents

PROTOCOL SUMMARY **4**
1 BACKGROUND **5**
1.1 Background Information 5
1.2 Rationale 6
1.3 Risk/Benefits 6
1.4 Study Conduct 7
1.5 Population 7
2 STUDY OBJECTIVES **7**
3 STUDY DESIGN **7**
3.1 Primary Study Endpoints/Secondary Endpoints 7
3.2 Study Design/Type 7
3.3 Duration 8
4 SELECTION AND WITHDRAWAL OF PARTICIPANTS **8**
4.1 Inclusion Criteria for Residents 8
4.2 Exclusion Criteria for Residents 8
4.3 Inclusion Criteria for Health care workers 8
4.4 Exclusion Criteria for Health care workers 8
4.5 Participant Withdrawal 8
5 STUDY INTERVENTION/INVESTIGATIONAL PRODUCT **9**
5.1 Study Product Description 9
5.2 Dosage, Preparation and Administration of Study Intervention/Investigational Product 9
5.3 Modification of Study Intervention/Investigational Product for a Participant 9
5.4 Accountability Procedures for the Study Intervention/Investigational Product 10
6 INFORMED CONSENT FOR RESIDENTS **10**
6.1 Resident Recruitment 10
6.2 Informed Consent Process for Residents 10
7 INFORMED CONSENT FOR HEALTH CARE WORKERS **11**
8 STUDY PROCEDURES/EVALUATIONS **12**
8.1 Clinical Evaluations for Resident Participants 12
8.2 Clinical Evaluation of Health Care Worker- Residents Participant Interactions 12
8.3 Laboratory Evaluations 13
9 UNANTICIPATED PROBLEMS AND SERIOUS ADVERSE EVENTS **13**
9.1 Adverse Events and their Grading 13
9.2 Attribution of Adverse Events 14
9.3 Adverse Event Reporting 14
10 QUALITY CONTROL AND QUALITY ASSURANCE **14**
11 STATISTICAL PLAN **14**
11.1 Statistical Methods 14
11.2 Sample size 15
12 ETHICAL CONSIDERATIONS **15**
13 DATA COLLECTION, HANDLING AND STORAGE **16**
14 REFERENCES **17**

Protocol Summary

Title: Human Microbiome and Health Care Associated Infection – Nursing Home Dwelling Older Veterans

Population: Approximately 25 older nursing home residents from a single geographic location in the US to achieve a final sample size of up to 20

Site: VA Maryland Health Care System Community Living Centers

Study Duration: Up to 24 months

Duration of Participant's Participation: Up to 52 weeks

Objectives:

- To collect specimens from multiple body sites in a non-invasive manner to serve as sources of measurement of the core microbiomes associated with the anterior nares, posterior pharynx, skin over the right subclavian vein, skin over the right femoral vein and the skin around the anus in older nursing home residents
- To collect specimens from multiple body sites in a non-invasive manner to serve as sources of measurement of the change in the core microbiomes associated with the anterior nares, posterior pharynx, skin over the right subclavian vein, skin over the right femoral vein and the skin around the anus in response to application of a decolonization regimen in older nursing home residents
- To collect specimens from the protective gowns and gloves of health care workers during routine care interactions with older nursing home residents to measure the impact of the decolonization regimen on this surrogate measure of transmission

1 Background

1.1 Background Information

Our adult bodies harbor ~10 times more microbial cells (e.g. bacteria, fungi) than human cells. Collectively these microbial cells are known as the human microbiome. A significant number of these microbes are difficult to grow in culture leading to inherent biases in the use of culture based methods to describe it. Our microbiome carries out many metabolic reactions not encoded for in the human genome and are necessary for our health. The NIH funded Human Microbiome Project (HMP) was developed to "to understand the microbial components of the human genetic and metabolic landscape and how they contribute to normal physiology and predisposition to disease" (1). The HMP is characterizing the microbiome of the gastrointestinal tract, the oropharynx, the anterior nares, the vagina and the skin in young healthy adults. A number of papers have begun to characterize the microbiome of the skin and anterior nares. Together they have shown that individuals vary less over time than they vary from others (2-5). Grice et al sampled the nares of 10 healthy young adults as a part of their skin survey (4). The nares contained predominantly Actinobacteria (Proprionobacteria ssp, Corynebacteria spp.), Firmicutes (Staphylococcus spp., Lactobacillus spp.), Proteobacteria (Gram-negatives) and Bacteroidetes in descending order of rank abundance. Most recently Costello et al. sampled multiple skin body sites at four time points from 7-9 healthy adults. They demonstrated that Gram-negative bacteria which come from the Proteobacteria phylum are present and their 16S rRNA sequences are present (<=10% of 16S rRNA sequences) in the human nose.

Bacteria and fungi of our microbiome also play an important role in the development of healthcare associated infections (HAI). These are infections that occur as complications of healthcare for other medical problems. The CDC estimates there are 1.7 million healthcare associated infections in the United States each year and almost 100,000 associated deaths (6). Hospital infection control programs strive to prevent healthcare associated infections. Recently forces external to individual hospitals have increased the pressure to prevent infections (7). Medicare will no longer reimburse for certain healthcare associated infections. Many states have made healthcare associated infections publicly reportable (8). This has led to the growing use of decolonization regimens.

"Decolonization" is a rapidly growing and yet controversial strategy to prevent the transmission of pathogens and healthcare-associated infections. The goal of decolonization is to prevent infections in the decolonized patients as well as reduce the risk of transmission of microorganisms to other patients. Decolonization involves the application of targeted or non-targeted antimicrobials to the skin and mucosal surfaces. Two commonly used agents for decolonization are intranasal mupirocin and topical chlorhexidine. Two recent meta-analyses concluded that mupirocin decreased *S. aureus* infections in *S. aureus* colonized surgical patients (9, 10). Intranasal mupirocin is increasingly being used to treat methicillin-resistant *S. aureus* (MRSA) colonization which is detected on mandated surveillance cultures (11). Chlorhexidine is used in intensive care units (ICU's) to prevent central line associated blood stream infections (12). Chlorhexidine baths have also been shown to decrease MRSA and VRE acquisition in the ICU setting (13).

1.2 Rationale

Given the role of our normal flora in serving as a barrier to colonization with more pathogenic organisms (aka colonization pressure), decolonization regimens could have unintended negative consequences. Our short term goal is to determine if these increasingly used decolonization regimens, targeted at controlling MRSA in particular, could result in a secondary negative effect of promoting colonization with pathogenic Gram-negative bacilli. We will also assess the impact of the decolonization regimen on a surrogate measure of transmission to validate the surrogate measure. The long term goal of this research is to use the information gained to develop new ways to manipulate the human microbiome to reduce the risk of healthcare associated infections with minimized negative consequences.

1.3 Risk/Benefits

The risks of the proposed study are minimal. There are possible risks and discomforts from this study for resident participants.

BACTROBAN NASAL (mupirocin calcium ointment, 2%) is a licensed, widely used, and generally well tolerated topical antibiotic available by prescription. Reported side effects noted in the packet insert include: Headache, 9%; Rhinitis, 6%; Respiratory disorder, including upper respiratory tract congestion, 5%; Pharyngitis, 4%; Taste perversion, 3%; Burning/stinging, 2% ; Cough, 2% and Pruritus, 1%. These side effects are temporary and stop after mupirocin is discontinued.

SAGE® 2% CHLORHEXIDINE GLUCONATE* CLOTH is an FDA- approved formulation and application for topical 2% chlorhexidine, an antiseptic solution which is available without a prescription. It is widely used for pre-operative skin cleansing and extremely well tolerated. Mild skin irritation can occur; this stops after chlorhexidine is stopped.

The sampling methods for the anterior nares, throat and skin sites is physically non-invasive, but there could some discomfort during the procedure. The potential discomfort from obtaining the specimens does not place any participant at physical risk. While taking the throat culture, the participant may gag and possibly vomit. A trained research coordinator will use very gentle pressure while performing the cultures to minimize these risks. However, there is the possibility of psychological discomfort from the specimen collection.

There is a risk that health information could be in accidentally disclosed to others outside the study. Confidentiality will be protected to the fullest extent permitted by law.

There may be adverse events that are not yet known.

There are few, if any potential risks to participant health care workers. They will wear disposable gowns and non- latex gloves while providing care for the enrolled residents. Wearing gowns and gloves are part of Standard (infection control) Precautions for certain care activities and offer no risk to the health care workers. Specimens will be taken from the disposable gown and gloves after care is provided and offer no risk to the health care workers. We are requesting a waiver of written documentation of informed consent, as this is the only document which would link the health care workers to the research study. We will not collect any individual data on the enrolled health care workers and thus there is no risk of loss of confidentiality.

There are no direct benefits to either group of study participants. Resident participants will receive a regimen that will eradicate *S. aureus* and other bacterial colonization in the short term. Although *S. aureus* colonization contributes to the risk of *S. aureus* infection, the absolute risk of *S. aureus* infection is small enough that this is not felt to be a direct benefit. This study will contribute knowledge about the human microbiome. *S. aureus* and other opportunistic pathogens are members of this microbiome. Understanding the human microbiome in *S. aureus* colonized participants can help us develop new ways to prevent *S. aureus* and other infections.

1.4 Study Conduct

This study will be conducted in compliance with the protocol approved by the UMB Institutional Review Board (hereafter referred to as IRB) and VAMHCS R&D Committee and according to Good Clinical Practice standards and the VHA Handbook 1200.05 Requirements for the Protection of Human Subjects in Research. No deviation from the protocol will be knowingly implemented without the prior review and approval of the IRB except where it may be necessary to eliminate an immediate hazard to a research participant. In such case, the deviation will be reported to the IRB as soon as possible.

1.5 Population

Study participants will be long term care (LTC) residents and the health care workers providing direct care to participating residents. All resident participants will be older adults (≥50 years old) that live in Community Living Centers in the VA Maryland HCS.

2 Study Objectives

This is a mechanistic patient-oriented research study designed to characterize the baseline microbiome of body sites associated with healthcare associated infection in nursing home dwelling older adults and to understand the effect of two commonly used local decolonization regimens on the microbiome of the nose, throat and three skin sites. In addition, the impact of the local decolonization regimen on the transmission of bacteria from participants to the gowns and gloves of health care workers during routine care will be assessed.

3 Study Design

3.1 Primary Study Endpoints/Secondary Endpoints

When examining the baseline microbiome as a whole, characterizing the bacteria present is the ‘endpoint’. The study will also look at changes to the baseline microbiome following decolonization regimens. A secondary endpoint is the transmission of bacteria from the study participant to the disposable gown or gloves of health care workers during observed episodes of care before and after the decolonization regimen.

3.2 Study Design/Type

This is a single-center mechanistic study to describe the microbiome of up to 25 older adults sequentially over a 1 year period, both before and after administration of topical antiseptic agent (chlorhexidine) and if *S. aureus* colonized in the anterior nares, intranasal antimicrobial ointment (mupirocin).

3.3 Duration

Study participants will have a total of 8 sets of non-invasive cultures from the anterior nares, posterior pharynx, skin over the subclavian vein, skin over the femoral vein, and the skin around the anus over a period of approximately 14 weeks plus one 12 month visit (+/- 3 months).

4 Selection and Withdrawal of Participants

4.1 Inclusion Criteria for Residents

- Age 50 years or older.
- Living in a participating nursing home for at least 80% of the past 3 months
- Willing to provide anterior nares, posterior pharynx, skin and throat specimens over the study period.
- Willing to use intranasal mupirocin and topical chlorhexidine over a five day period.
- Provide signed and dated informed consent from subject or LAR.

4.2 Exclusion Criteria for Residents

- Recent history of MRSA colonization
- Use of mupirocin nasal ointment used in past 3 months
- Use of topical chlorhexidine used in past 3 months
- History of an allergic reaction to chlorhexidine or mupirocin

4.3 Inclusion Criteria for Health care workers

- Health care worker at study site
- Has direct interaction with participating residents at study site
- Willing to wear disposable gown and gloves during direct interaction with participating resident
- Verbal informed consent

4.4 Exclusion Criteria for Health care workers

- None

4.5 Participant Withdrawal

Study participants will be withdrawn from the study if found to have been ineligible on enrollment. No follow-up with withdrawn participants is required. Resident participants will not be withdrawn for acute care hospitalization or receipt of antimicrobial agents during the study period. They will continue to be followed throughout the study as a ‘natural’ experiment, but their post-antibiotic or hospitalization data will be excluded from the primary analysis.

5 Study Intervention/Investigational Product

5.1 Study Product Description

5.1.1 Acquisition

BACTROBAN NASAL ointment (mupirocin calcium ointment, 2%) and SAGE 2% CHLORHEXIDINE GLUCONATE CLOTHS will be dispensed by the VAMHCS Investigational Pharmacy.

5.1.2 Formulation, Packaging, and Labeling

The study products are BACTROBAN NASAL ointment (mupirocin calcium ointment, 2%) and SAGE 2% CHLORHEXIDINE GLUCONATE CLOTHS. BACTROBAN NASAL is a licensed medication packaged and labeled by GlaxoSmithKline. BACTROBAN NASAL (mupirocin calcium ointment, 2%) is packaged in 1.0-gram tubes and is for intranasal use only. SAGE 2% CHLORHEXIDINE GLUCONATE CLOTHS is an FDA-approved medication packaged and labeled by SAGE Products Inc. SAGE 2% CHLORHEXIDINE GLUCONATE CLOTHS is packaged with two 7.5in x 7.5in cloths to a package (3 sets of 2 cloth packages come bundled together).

5.1.3 Product Storage and Stability

BACTROBAN NASAL ointment (mupirocin calcium ointment, 2%) and SAGE 2% CHLORHEXIDINE GLUCONATE CLOTHS will be stored between 20-25°C (68-77°F), as directed by the manufacturers.

5.2 Dosage,

5.3 Preparation and Administration of Study Intervention/Investigational Product

Resident participants will be treated topically once daily with SAGE 2% CHLORHEXIDINE GLUCONATE CLOTHS topically on the five days before study visit #3. The cloths can be applied by the participant or study staff or nursing staff. Participants that are colonized with *S. aureus* will also be treated twice daily with BACTROBAN NASAL ointment (mupirocin calcium ointment, 2%) intranasally on days -5, -4,-3, -2, and -1 before study visit #3. The ointment can be applied by the participant or study staff or nursing staff.

5.4 Modification of Study Intervention/Investigational Product for a Participant

If a resident participant develops allergic symptoms or other signs of intolerance to SAGE 2% CHLORHEXIDINE GLUCONATE CLOTHS or BACTROBAN NASAL, that medication will be stopped. Make up of missed doses will be at the discretion of the PI and not be considered a deviation.

5.5 Accountability Procedures for the Study Intervention/Investigational Product

The PI is responsible for ensuring that a current record of product disposition is maintained and product is dispensed only at an official study site by authorized personnel as required by applicable regulations and guidelines. SAGE 2% CHLORHEXIDINE GLUCONATE CLOTHS and BACTROBAN NASAL ointment (mupirocin calcium ointment, 2%) will be dispensed by the VAMHCS Investigational Pharmacy, and subsequently distributed to the participating nursing home s. The resident participants will receive the products via the nursing staff at the Community Living Centers. Documentation of all products received, dispensed and destroyed will be kept by the research pharmacist.

6 Informed Consent for Residents

6.1 Resident Recruitment

We are requesting a HIPAA waiver to allow the study coordinator to review the medical records of potential resident participants to determine initial eligibility, medical decision making capacity and contact information for the next of kin or legally authorized representative via the VA's Computerized Patient Record System (CPRS). Accessing this information from the resident's medical record reduces the burden on the resident with regard to time and research procedures, increases the feasibility of the study, and does not confer any additional risk.

We will distribute an IRB approved informational flier about the study to eligible residents and provide a phone number for follow-up. We will also present at any family meetings to explain study procedures, answer questions and address concerns. A study coordinator will approach eligible residents about participation using an IRB approved script.

6.2 Informed Consent Process for Residents

The study coordinator will tell the resident about the study using an IRB approved script. If the resident expresses interest in the study and is willing to participate, the study coordinator will verbally review the information on the IRB approved informational flier and the informed consent form with the resident in a private area. The resident will be asked if he/she wishes for the study coordinator to read the informed consent form verbatim or to summarize it as the resident follows along.

Because cognitive impairment is common in the nursing home population, the resident will be evaluated for his/her ability to give informed consent. The resident must be alert and able to communicate in order to give informed consent. He /she will be asked the following questions before informed consent to participant is considered to have occurred: "Why are we doing the study? Does the study have any risks? What are you going to do in the study? Do you have to participate in the study? If you don't want to be in the study anymore, what do you do?" as described in Evaluation to Sign Consent. If the potential participant is unable to answer four of these five questions correctly after two opportunities, he/she is deemed unable to consent and his/her next of kin as listed in the medical record will decide whether it is in his/her best interest to participate in the study.

When a resident lacks the capacity to make the decision to participate in the study, his/her next of kin as listed in the emergency contact section of CPRS will decide whether it is in his/her

best interest to participant in the study if the emergency contact has the authority to act as the resident's legally authorized representative (LAR) per VHA Handbook 1200.05, paragraph 36c.

The LAR will be contacted and will receive: a letter based on the informational flier for residents, the informed consent form, and the HIPAA authorization form. The LAR will be given the same opportunity to have his/her questions answered.

The resident or LAR will sign his/her name on the study's copy of the Consent and HIPAA Forms. If a resident is unable to sign his/her name, he/she will make an X and a non-study related witness will also sign. Resident or LAR will receive a copy of the signed consent forms.

Participant assent to study procedures will be obtained prior to any procedures for those residents that are deemed unable to consent for themselves and consent of an LAR is obtained. Prior to study procedures, the study coordinator will briefly explain the study purpose (degree of explanation will depend on the resident's level of cognition) and study procedures (Assent Script). Participant will assent by verbal agreement or positive gesture or lack of dissent if unable to provide verbal agreement or positive gesture. If resident does not assent to study procedures, no further procedures will be performed.

7 Informed Consent for Health care workers

7.1 Recruitment Process

Health care workers will be recruited through participating Community Living Centers. Prior to study commencement at a CLC, a meeting will be scheduled with facility/unit administrators including the Medical Director and Director of Nursing at each CLC. Upon confirming their agreement to participate, we will meet with unit nurse managers and providers, distribute an informational flier to all health care workers at that facility that interact with residents and provide a phone number for follow- up. We will also present at a staff meeting to explain study procedures, answer questions and address concerns. In order to assure that health care workers are not coerced into participating, we will not report participation to supervisors. There will be no penalties or loss of benefits for not participating.

7.2 Informed Consent Process for Health Care Workers

The health care worker will be told about the study by the study coordinator following a specific script for informed consent. If the health care worker is willing to participate, study staff will provide a copy of the script for informed consent and a handout for informed consent. Study staff will ask the following questions to assure that informed consent has been obtained.

"What is the purpose of the study; i.e., what are we attempting to learn from it? What is your role in this study? Are you required to participate in this study, or is your participation voluntary? What will you gain as a result of participation? Are there risks to participating?" Study staff will review the answers to any incorrect responses.

The health care worker will not be required to sign the informed consent document, as this is the only study document that will link the health care workers to the research study. We are requesting a waiver of written documentation of informed consent to protect confidentiality in this vulnerable population. At each study health care worker-resident interaction, the study coordinator will confirm continued consent.

8 Study Procedures/Evaluations

All visits will occur in the Community Living Centers of the VA Maryland HCS.

8.1 Clinical Evaluations for Resident Participants

Enrollment/Study Visit #1: Informed consent will be obtained prior to any Study Visit #1 data collection and procedures. . At the first visit, demographic information and a brief medical history are obtained by review of CPRS. The brief medical history includes current and recent medications and past medical and surgical history. The study coordinator will complete a brief physical assessment of the culture sites and obtain non-invasive specimens of the following sites: anterior nares, posterior pharynx, skin over the femoral vein, skin over the subclavian vein, and the skin around the anus.

Study Visit #2: A focused medical history, a review of concomitant medications from CPRS, and a brief physical assessment of their anterior nares, posterior pharynx and skin sites will be performed. Participants will be informed of their Staph aureus culture results from the first nose culture that was done. Specimens from the anterior nares, posterior pharynx, and skin sites will be obtained. Study medications will be dispensed and used as described above.

Study Visits #3, #4, #5, #6, #7 and #8: A focused medical history, a review of concomitant medications from CPRS, and a brief physical assessment of their anterior nares, posterior pharynx and skin sites will be performed. Specimens from the anterior nares, posterior pharynx, and skin sites will be obtained.

The visit time points represent ideal visit times, but this exact timing is not required. We will be using the following guidelines:

1) The first seven visits must occur no more than 11 weeks after enrollment.
2) Resident participants should have 2 sets of cultures collected in the 2 weeks prior to administration of chlorhexidine/mupirocin. These initial 2 sets of cultures can occur no closer than 10 days apart.
3) Resident participants should have 4 sets of cultures collected in the 4 weeks after chlorhexidine/mupirocin administration. These 4 sets of cultures can occur no closer than 5 days apart.
4) Resident participants should have a second to last set of cultures collected after the 4 weekly set of cultures. This second to last set of cultures can occur no closer than 2 weeks after the last weekly culture.
5) Resident participants should have a last set of cultures collected 1 year +/- 3 months after the enrollment visit.

Cultures not collected at the designated time or missed altogether will not be reported as deviations. Missed visits will not be reported as deviations. For the purposes of this study, *S. aureus* colonization will be defined as a positive culture from study visit #1.

8.2 Clinical Evaluation of Health Care Worker- Residents Participant Interactions

Each study interaction will consist of an observation of care or resident-health care workers interaction. There will be a minimum of 6 and a maximum of 25 per resident prior to

medication administration and during medication administration plus 1 week. Missed visits will not be reported as deviations. The types of care and time of care provided to the resident by health care workers will be recorded. Health care workers will be provided with a disposable gown and gloves to wear during each episode of care. The types of care and duration of care will be recorded at each follow up visit. The gown and gloves will be cultured immediately after the health care workers are done providing care.

8.3 Laboratory Evaluations

During each study visit, a specimen is obtained by study coordinator from each of the following sites: anterior nares, posterior pharynx, skin over the femoral vein, skin over the subclavian vein, and the skin around the anus. These specimens are cultured for *S. aureus* and gram-negative bacilli in the UM SOM Research Microbiology Laboratory and are also analyzed for the presence of all bacteria using metagenomic techniques in the UM SOM Institute for Genome Sciences. During study interactions, specimens are obtained from the gowns and gloves of health care workers and tested for bacteria in the UM SOM Research Microbiology Laboratory. Study specimens that remain after study-specific procedures will be banked at the Baltimore VA Medical Center for future studies if participants consent to future studies.

9 Unanticipated Problems and Serious Adverse Events

9.1 Adverse Events and their Grading

An ADVERSE EVENT (AE) is any unfavorable, harmful or pathological change in a research participant as indicated by physical signs, symptoms and/or clinically significant laboratory abnormalities that occur in association with the study, whether or not considered study-related. This definition includes intercurrent illnesses, injuries, and exacerbation of preexisting conditions. Stable preexisting conditions and elective procedures to address such conditions are not adverse events.

A SERIOUS ADVERSE EVENT is any adverse experience that results in any of the following outcomes: Death, a life-threatening adverse drug experience, inpatient hospitalization or prolongation of existing hospitalization, a persistent or significant disability/incapacity, or a congenital anomaly/birth defect. Important medical events that may not result in death, be life-threatening, or require hospitalization may be considered a serious adverse drug experience when, based upon appropriate medical judgment, they may jeopardize the patient or participant and may require medical or surgical intervention to prevent one of the outcomes listed in this definition.

The severity of the adverse events will defined as follows:

0 - No adverse event or within normal limits

1 - Mild - awareness of sign, symptom or event but easily tolerated

2 - Moderate - discomfort enough to cause interference in usual activity and may warrant intervention

3 - Severe - incapacitating in ability to do usual activities or significantly affects clinical status, and warrants intervention

4 - Life threatening or disabling AE

5 - Fatal AE

9.2 Attribution of Adverse Events

The Principal Investigator will determine attribution of adverse events. The following scale will assess the relationship of AEs to the test procedure/device/agent:

Not related: no temporal association, or the cause of the event has been identified, or the drug cannot be implicated

Possibly related: temporal association, but other etiologies are likely to be the cause; however involvement of the drug cannot be excluded

Probably related: temporal association, other etiologies are possible but unlikely

Related: established temporal or other association for event not reasonably explained by the patient's known clinical state or any other factor

9.3 Adverse Event Reporting

Serious Adverse Events that are probably or definitely related to the research will be reported within 48 hours of the investigator becoming aware of the SAE to the independent data safety monitor, UMB IRB according to established IRB policy and to the VAMHCS Research and Development Committee. The principal investigator will evaluate the adverse event and determine whether the adverse event affects the Risk/Benefit ratio of the study and whether modification to the protocol or consent form are required.

The principal investigator will conduct a review of all adverse events every 12 months. They will evaluate the frequency and severity of the adverse events and determine if modifications to the protocol or consent form are required. This summary of the adverse events will be reported to the UMB IRB annually, when renewal is sought.

10 Quality Control and Quality Assurance

The PI and affiliated institution are responsible for conducting the study in compliance with the IRB-approved protocol, all applicable federal and state regulations, and the International Conference on Harmonization, E6 Good Clinical Practice by adhering to the requirements for collecting, documenting and reporting complete and accurate data. A separate Data Quality Assurance (QA) Plan will be developed for this protocol to describe the frequency of review of study related data and the roles and responsibilities for activities related to Quality Control (QC).

11 Statistical Plan

11.1 Statistical Methods

11.1.1 Analysis Plan for Microbial Community Profiling

In this project, we will utilize model-based statistical methods to study the dynamics and composition of the microbial communities present in the anterior nares, posterior pharynx and two skin sites in two populations (from Protocol HP-48003 Microbiome and Health Care Associated Infection – Nursing Home Dwelling Older Adults and Protocol HP-47561 Community dwelling Older Adults and this protocol Nursing Home dwelling Older Adults) with and without current exposure to the healthcare environment, and before and after decolonization using treatment with topical chlorhexidine and intranasal mupirocin if *S. aureus* colonized. Data from this protocol will be coded before being combined with the non-VA data. These analyses will be performed in association with the various metadata collected and will be critical to be able to answer the various questions associated with our project, which include:

1. Over time within individuals and at a) nose, b) throat, c) skin sites, what are the microbial communities present, their relative/absolute abundance?
2. Are there differences between individuals at a) nose, b) throat, c) skin sites by living environment (community dwelling vs. nursing home dwelling)?
3. Are there differences among individuals, by *S. aureus* colonization status, age groups and sex?
4. What are the changes, within individuals, in the microbiome associated with decolonization over time at a) nose, b) throat, c) skin sites, in term or composition and relative/absolute abundance?
5. What are the changes in the microbiome of each individual from the baseline to after decolonization at a) nose, b) throat, c) skin sites, in relation to the decolonization regimen (chlorhexidine vs. chlorhexidine plus mupirocin) and living environment (community dwelling vs. nursing home dwelling)?

11.1.2 Analysis Plan for Microbiologic Data

The goals of the microbiological analysis are two-fold. The first goal is descriptive to determine which decolonization regimen will be used and to document the effectiveness of the two regimens in reducing colonization with *S. aureus* and pathogenic Gram-negative bacilli at the different body sites sampled. In addition we will assess changes in the transmission of bacteria to the gowns and gloves of health care workers from the individual from the baseline to after decolonization. The second goal will compare the effectiveness of the two regimens in the two different populations: community dwelling and nursing home dwelling older adults.

11.2 Sample size

How many subjects (or specimens, or charts) will be used in this study?
Local: 25 Worldwide: 25

We plan on enrolling 25 subjects to end up with a final sample size of 20. Sample sizes were chosen based on a "worst case scenario" estimate of sample size, feasibility and cost. Unlike more traditional genetic association studies, where theoretical sample calculations can be performed, the current study uses more novel data mining approaches applied to data obtained from high-throughput genotyping technology, in which many different potential OTUs are compared between groups (pre and post decolonization within a subject). Currently, there is no generally accepted theoretical power calculations for the analyses proposed here. Since the subjects will serve as their own controls (pre and post decolonization), we increase power to detect significant differences and potentially avoiding confounding factors (6). Indeed, the kind of novel statistical analytical methods we propose to employ in this study have been empirically shown to have higher power than traditional methods to detect significant trends (7-9). These results allow us to use sample size calculations based on traditional methods as a "worst case scenario" estimate, assuming the power using such novel approaches will be higher.

12 Ethical Considerations

This study will be conducted according to US and international standards of Good Clinical Practice (FDA regulations 21 CFR 312 for IND studies and FDA guidance E6) for all studies. Applicable government regulations and UMB research policies and procedures will also be followed.

All subjects for this study will be provided a consent form describing this study and providing sufficient information for subjects to make an informed decision about their participation in this study. This consent form will be submitted with the protocol for review and approval by the IRB. The formal consent of a subject, using the IRB-approved consent form, will be obtained before that subject is submitted to any study procedure. This consent form must be signed by the subject, and the investigator-designated research professional obtaining the consent.

13 Data Collection, Handling and Storage

The Principal Investigator is responsible to ensure the accuracy, completeness, legibility, and timeliness of the data reported. Data collection is the responsibility of the study staff. All study personnel will be current in Information Security Training (VHA Privacy and Information Security and Rules of Behavior Training).

Paper records or case report forms will be filled out at the participating VA Community Living Centers or in the research team offices. Copies of the paper records or case report forms will serve as source documents and maintained for recording data for each subject enrolled in the study. All source documents will be completed in a legible manner to ensure accurate interpretation of data. Black ink is required to ensure clarity of reproduced copies. When making changes or corrections, the original entry will be crossed out with a single line, and the change initialed and dated. Erasing, overwriting, or use of correction fluid or tape will not be done.

All source documents and laboratory reports will be reviewed by the clinical team and data entry staff, who will ensure that they are accurate and complete. AEs must be graded, assessed for severity and causality, and reviewed by the site PI or designee.

Confidentiality will be maintained to the fullest extent permitted by law. Research records generated in this study will be transported from clinical protocol study site to data entry site by study personnel, accompanied by an Authorization to Transport and Utilize VA Sensitive Information Outside Protected Environments. Data will be maintained on a secure electronic central database on a VA research server (vhabalrsch). Identifiable electronic study data will never be removed from behind the VA firewall. Data will be backed up according to the VA network back up schedule. Paper records will be stored in locked filing cabinets behind a locked door in office space at the University of Maryland Baltimore that is accessible to the study PI. Study personnel who leave the research team will have their access to study data removed immediately. If data is lost/stolen, the VAMHCS, ISO/PO/PI and the UMB IRB will be notified immediately.

A unique identifier or code will be assigned to each resident participant. Bacterial isolates and culture specimens and datasets used for analysis will be labeled with this code instead of the subject name or last 4 of SSNO. Only the study personnel who directly interact with subject or manage the subject‘s clinical protocol data will have access to participant identifying information. Culture data will be entered directly into the relational database; however, laboratory personnel will not have access to participant identifiers in the database.

The Principal Investigator will maintain all records pertaining to this study according to Records Control Schedule 10-1 (indefinitely). As soon as permitted and when data collection is complete - source documents will be shredded and identifying information (names/last 4 of SSNO) will be removed from the database. During the entire study, all data will be managed centrally at the Baltimore VA Medical Center. There will be a single data table which maintains the link between the unique code and patient identifiers. This table will be maintained on a VA research server (vhabalrsch). As soon as permitted and when data collection is complete, the link

between code and identifying information will be deleted on the server and in any backups. If any paper copies of the link have been made, they will be shredded.

14 References

1. Turnbaugh PJ, Ley RE, Hamady M, Fraser-Liggett CM, Knight R, Gordon JI. The human microbiome project. *Nature.* 2007; 449(7164): 804-810. [PMID: 17943116].
2. Fierer N, Hamady M, Lauber CL, Knight R. The influence of sex, handedness, and washing on the diversity of hand surface bacteria. *Proc Natl Acad Sci U S A.* 2008; 105(46): 17994-17999. [PMID: 19004758].
3. Gao Z, Tseng CH, Pei Z, Blaser MJ. Molecular analysis of human forearm superficial skin bacterial biota. *Proc Natl Acad Sci U S A.* 2007; 104(8): 2927-2932. [PMID: 17293459].
4. Grice EA, Kong HH, Conlan S, Deming CB, Davis J, Young AC, Bouffard GG, Blakesley RW, Murray PR, Green ED, Turner ML, Segre JA. Topographical and temporal diversity of the human skin microbiome. *Science (New York, N Y.* 2009; 324(5931): 1190-1192. [PMID: 19478181].
5. Costello EK, Lauber CL, Hamady M, Fierer N, Gordon JI, Knight R. Bacterial community variation in human body habitats across space and time. *Science.* 2009 Dec 18; 326(5960): 1694-1697. [PMID: 19892944].
6. Klevens RM, Edwards JR, Richards CL,Jr., Horan TC, Gaynes RP, Pollock DA, Cardo DM. Estimating health care-associated infections and deaths in U.S. hospitals, 2002. *Public Health Rep.* 2007; 122(2): 160-166. [PMID: 17357358].
7. Arias KM. Mandatory reporting and pay for performance: Health care infections in the limelight. *AORN J.* 2008; 87(4): 750-758. [PMID: 18395020].
8. Meier BM, Stone PW, Gebbie KM. Public health law for the collection and reporting of health care-associated infections. *Am J Infect Control.* 2008; 36(8): 537-551. [PMID: 18926306].
9. van Rijen M, Bonten M, Wenzel R, Kluytmans J. Mupirocin ointment for preventing staphylococcus aureus infections in nasal carriers. *Cochrane Database Syst Rev.* 2008(4): CD006216. [PMID: 18843708].
10. Ammerlaan HS, Kluytmans JA, Wertheim HF, Nouwen JL, Bonten MJ. Eradication of methicillin-resistant staphylococcus aureus carriage: A systematic review. *Clin Infect Dis.* 2009; 48(7): 922-930. [PMID: 19231978].
11. Robicsek A, Beaumont JL, Paule SM, Hacek DM, Thomson RB,Jr., Kaul KL, King P, Peterson LR. Universal surveillance for methicillin-resistant staphylococcus aureus in 3 affiliated hospitals. *Ann Intern Med.* 2008; 148(6): 409-418. [PMID: 18347349].
12. Bleasdale SC, Trick WE, Gonzalez IM, Lyles RD, Hayden MK, Weinstein RA. Effectiveness of chlorhexidine bathing to reduce catheter-associated bloodstream infections in medical intensive care unit patients. *Arch Intern Med.* 2007; 167(19): 2073-2079. [PMID: 17954801].
13. Climo MW, Sepkowitz KA, Zuccotti G, Fraser VJ, Warren DK, Perl TM, Speck K, Jernigan JA, Robles JR, Wong ES. The effect of daily bathing with chlorhexidine on the acquisition of methicillin-resistant staphylococcus aureus, vancomycin-resistant enterococcus, and healthcare-associated bloodstream infections: Results of a quasi-experimental multicenter trial. *Crit Care Med.* 2009; 37(6): 1858-1865. [PMID: 19384220].
14. Bode LG, Kluytmans JA, Wertheim HF, Bogaers D, Vandenbroucke-Grauls CM, Roosendaal R, Troelstra A, Box AT, Voss A, van der Tweel I, van Belkum A, Verbrugh HA, Vos MC. Preventing surgical-site infections in nasal carriers of staphylococcus aureus. *N Engl J Med.* 2010 Jan 7; 362(1): 9-17. [PMID: 20054045].

15. Harris AD, McGregor JC, Johnson JA, Strauss SM, Moore AC, Standiford HC, Hebden JN, Morris JG,Jr. Risk factors for colonization with extended-spectrum beta-lactamase-producing bacteria and intensive care unit admission. *Emerging Infect Dis.* 2007; 13(8): 1144-1149.
16. Thom KA, Johnson JA, Strauss SM, Furuno JP, Perencevich EN, Harris AD. Increasing prevalence of gastrointestinal colonization with ceftazidime-resistant gram-negative bacteria among intensive care unit patients. *Infect Control Hosp Epidemiol.* 2007; 28(11): 1240-1246.
17. Selden R, Lee S, Wang WL, Bennett JV, Eickhoff TC. Nosocomial klebsiella infections: Intestinal colonization as a reservoir. *Ann Intern Med.* 1971 May; 74(5): 657-664. [PMID: 5559431].
18. Hidron AI, Edwards JR, Patel J, Horan TC, Sievert DM, Pollock DA, Fridkin SK. NHSN annual update: Antimicrobial-resistant pathogens associated with healthcare-associated infections: Annual summary of data reported to the national healthcare safety network at the centers for disease control and prevention, 2006-2007. *Infect Control Hosp Epidemiol.* 2008; 29(11): 996-1011. [PMID: 18947320].
19. Schloss PD, Handelsman J. Introducing DOTUR, a computer program for defining operational taxonomic units and estimating species richness. *Appl Environ Microbiol.* 2005 Mar; 71(3): 1501-1506. [PMCID: PMC1065144] [PMID: 15746353].
20. Lozupone C, Knight R. UniFrac: A new phylogenetic method for comparing microbial communities. *Appl Environ Microbiol.* 2005 Dec; 71(12): 8228-8235. [PMCID: PMC1317376] [PMID: 16332807].
21. Ravel J, Gajer P, Abdo Z, Schneider GM, Koenig SS, McCulle SL, Karlebach S, Gorle R, Russell J, Tacket CO, Brotman RM, Davis CC, Ault K, Peralta L, Forney LJ. Microbes and health sackler colloquium: Vaginal microbiome of reproductive-age women. *Proc Natl Acad Sci U S A.* 2010 Jun 3 [PMID: 20534435].
22. Schloss PD, Handelsman J. Introducing SONS, a tool for operational taxonomic unit-based comparisons of microbial community memberships and structures. *Appl Environ Microbiol.* 2006 Oct; 72(10): 6773-6779. [PMCID: PMC1610290] [PMID: 17021230].
